# Comparing the effectiveness of different vaccines in individuals with and without autoimmune diseases: a study protocol for a target trial emulation

**DOI:** 10.3389/fpubh.2025.1583434

**Published:** 2025-05-26

**Authors:** Robin Denz, Heike van de Sand, Jale Basten, Katharina Meiszl, Marianne Tokic, Theresa Oganowski, Thomas Grüter, Stephanie Stock, Dusan Simic, Arim Shukri, Uta Kiltz, Maria Zacharopoulou, Horst Christian Vollmar, Ina Carola Otte, Chantal Giehl, Romy Lauer, Anastasia Suslow, Andreas Stallmach, Anika Franz, Ursula Marschall, Joachim Saam, Catharina Schumacher, Katja Blaschke, Ingo Meyer, Kerstin Hellwig, Nina Timmesfeld, The VAC-MAC Consortium

**Affiliations:** ^1^Department of Medical Informatics, Biometry and Epidemiology, Ruhr University Bochum, Bochum, Germany; ^2^PMV Research Group, Medical Faculty and University Hospital Cologne, University of Cologne, Cologne, Germany; ^3^Department of Neurology, St. Josef Hospital, Ruhr University Bochum, Bochum, Germany; ^4^Department of Neurology and Stroke Unit, Evangelical Hospital Lippstadt, Lippstadt, Germany; ^5^Faculty of Medicine and University Hospital Cologne, Institute of Health Economics and Clinical Epidemiology (IGKE), University of Cologne, Cologne, Germany; ^6^Rheumazentrum Ruhrgebiet, Herne, Germany; ^7^Department of Rheumatology, Ruhr University Bochum, Bochum, Germany; ^8^Medical Faculty, Institute of General Practice and Family Medicine (AM RUB), Ruhr University Bochum, Bochum, Germany; ^9^Department of Geriatric Medicine, Marien Hospital Herne, Ruhr University Bochum, Herne, Germany; ^10^Department of Internal Medicine IV (Gastroenterology, Hepatology and Infectious Diseases), Jena University Hospital, Jena, Germany; ^11^Department Medicine and Health Services Research, BARMER Institute for Health System Research, Wuppertal, Germany

**Keywords:** claims data, multiple sclerosis, chronic inflammatory rheumatoid diseases, chronic inflammatory bowel diseases, vaccine effectiveness, target trial

## Abstract

**Aim:**

This article presents the study protocol of a retrospective cohort study designed to compare the effectiveness of herpes zoster, and influenza vaccines in individuals with multiple sclerosis (MS), chronic inflammatory bowel diseases (IBD), or chronic inflammatory rheumatic diseases (CIRD) to individuals without these diseases, using claims data of one of the largest population based health insurances in Germany.

**Background:**

Individuals with autoimmune diseases such as MS, IBD, and CIRD are more susceptible to vaccine preventable infectious diseases such as influenza and herpes zoster, due to the autoimmune disease itself, the presence of comorbidities and immunosuppressive therapies. Vaccines are the primary means to prevent such diseases. The efficacy of these vaccines is usually estimated using large randomized controlled trials, from which patients with MS, IBD, and CIRD are often excluded. It is therefore unclear whether these vaccines are also effective for these patients.

**Design:**

A target trial emulation based on observational claims data of a statutory health insurance company is proposed.

**Methods:**

This study will aim to emulate multiple target trials to compare the effectiveness of herpes zoster and influenza vaccines in patients with and without MS, IBD and CIRD using data from a large German statutory health insurance provider (BARMER). The primary outcome for each vaccine effectiveness analysis is the disease itself. The analysis will be carried out using both time-dependent matching and a multivariable Cox proportional hazards model in conjunction with g-computation. Additionally, the moderating effect of immunosuppressive therapies on the vaccine effectiveness will be estimated using a stratified secondary analysis.

**Discussion:**

This study will estimate and compare the effectiveness of influenza and herpes zoster vaccines in individuals with and without MS, IBD, and CIRD. Because of the large amount of data, this study will also be able to investigate the role of the immunosuppressive medication on vaccine effectiveness, which may provide guidance for vaccine administration guidelines.

## 1 Introduction

Individuals with autoimmune diseases, such as multiple sclerosis (MS), chronic inflammatory bowel diseases (IBD), or chronic inflammatory rheumatoid diseases (CIRD) have a highly increased chance of being infected with various vaccine-preventable infectious diseases (1, 2). This increased susceptibility, coupled with a higher chance of worse outcomes, has been shown repeatedly for MS patients (3), IBD patients (4, 5), and CIRD patients (6) compared to the general population and is thought to be the result of the autoimmune disease itself, existing comorbidities and the use of immunosuppressive therapies (5). Influenza and herpes zoster are two such diseases, which may lead to serious complications or even death (7, 8). The primary way to prevent these diseases in the general population is through the use of vaccines. The efficacy and effectiveness of these vaccines is usually determined using large randomized controlled trials, from which individuals with autoimmune diseases are often excluded (9). It is therefore unclear whether the effectiveness of the respective vaccines in individuals with autoimmune diseases is comparable to the effectiveness in individuals without these diseases.

Only a few studies tried to directly estimate the effects of herpes zoster or influenza vaccines in MS, IBD, or CIRD patients. Some single-arm prospective studies have shown an adequate immune response to an influenza vaccination in MS patients, but inconclusive results for pneumococcal vaccination in similar patients (10, 11). A few observational studies, based on routinely collected claims data, have demonstrated that vaccination against herpes zoster may be safe and effective in IBD patients (12–14), but the evidence for CIRD and MS is much more limited (15, 16). A meta-analysis of randomized controlled trials aiming to estimate the safety and effectiveness of the herpes zoster vaccine in both immunocompetent and immunocompromized patients in general also found the vaccine to be effective and safe (17), although it is unclear whether these results hold for MS or CIRD patients specifically.

This lack of evidence may be one reason why patients with autoimmune diseases are hesitant to get vaccinated, despite numerous recommendations and guidelines mentioning the benefits of vaccination (18–21). In this article we present a study protocol of a large-scale observational, retrospective study based on claims data from a large German statutory health insurance company that aims to compare the effectiveness of influenza and herpes zoster vaccines between individuals with and without MS, IBD, or CIRD. We first use the target trial framework to describe hypothetical randomized studies that we wish to emulate using the available data (22). Afterwards we give detailed explanations on how we will try to achieve this emulation. This includes a detailed description of inclusion criteria, definitions of treatments, outcomes and relevant confounders as well as an overview of the statistical analysis plan.

## 2 Methods and analysis

### 2.1 Aims and objectives

The primary aim of this study is to estimate the effectiveness of two different kinds of vaccines in individuals with either MS, IBD or CIRD and individuals without these diseases and to compare the effectiveness between these populations. More specifically, we are interested in the effectiveness of two types of vaccines:

1. Vaccines against herpes zoster (shingles).
2. Vaccines against influenza.

A secondary aim of this study is to investigate the impact of immunosuppressive medication taken before vaccination on vaccine effectiveness in each of the three disease groups. It has been shown that the use of immunosuppressive medication raises the risk of developing infectious diseases (23). Since a large proportion of MS, IBD and CIRD patients receive such medications, disentangling the effect of the actual disease and the medication is crucial to make valid vaccine recommendations (24).

### 2.2 Target trials and target estimand

To make the aims of our study as clear as possible we formulate multiple *target trials*, as proposed by Hernán and Robins (22). A target trial is a description of a hypothetical study that we, the investigators, would conduct if there were no ethical or practical limitations on what we could do. It should include a description of all relevant study characteristics, such as inclusion- and exclusion criteria, a clear definition of the population of interest and how the study would be conducted. The aim is then to *emulate* these trials using available data and appropriate analysis techniques (22). This framework makes it easier to follow the International Council for Harmonization of Technical Requirements for Pharmaceuticals for Human Use guidelines (ICH-E9) for defining target estimands (25, 26) and allows us to formulate a clear definition of the time of inclusion of each patient (“time zero”) (27). Similar studies concerned with COVID-19 vaccine effectiveness have also used this approach effectively (28). Table 1 summarizes the most important components of the respective target trial protocols.

**Table 1:** A summary of the most important protocol components for each of the planned target trial emulations.

**Protocol component**	**Target trial**	**Emulation with observational data**
Data Source	Prospectively collected data between 2013 and 2021.	Routinely collected claims data from a large German statutory health insurance company (BARMER) from 2013 to 2021.
Eligibility Criteria	(1) ≥ 18 years old (2) Biological sex of either male or female (3) No organ transplantation in the past 2 years (4) No cancer in the past 2 years (5) No autoimmune-related disease other than MS, IBD, or CIRD in the past 2 years (6a) For the herpes zoster trial: No previous herpes zoster vaccination (6b) For the influenza trials: No previous influenza vaccination in the current influenza season	Same as in the target trial, but with one additional criteria: (7) Enrollment in the BARMER health insurance company for ≥ 2 years with interruptions of no more than 5% of the total time
Treatment Strategies	(a) Vaccination against herpes zoster infection at treatment assignment with another vaccination between two and six months afterwards (b) Vaccination against influenza infection at treatment assignment (c) No vaccination at treatment assignment (no placebo) Treatment strategies (a) and (b) are compared to (c) in their separate trials.	
Treatment Assignment	Individual randomization, stratified on disease status (MS, IBD, CIRD or none of these).	To emulate randomization we adjust for multiple confounders using a combination of matching and g-computation.
Start and end of trial	(1) Herpes zoster vaccine effectiveness trial: 01.12.2018–31.12.2021^a^ (2) Influenza vaccine effectiveness trials:*^b^* (a) 01.09.2015–31.05.2016 (b) 01.09.2016–31.05.2017 (c) 01.09.2017–31.05.2018 (d) 01.09.2018–31.05.2019	
Start and end of follow-up	Start of follow-up: time of assignment to treatment strategy. End of follow-up: The earliest of death, loss-to-follow-up or administrative end-of-follow-up (end of the respective study).	
Outcomes	(1) Herpes zoster vaccine effectiveness: medically attended Herpes zoster infection (2) Influenza vaccine effectiveness: (a) medically attended influenza infection (b) severe medically attended influenza infection	
Causal contrast	(1) The ratio between the vaccine effectiveness (1 - the relative risk to develop the outcome at time *t* given the treatment) in the diseased group (individual has either MS, IBD or CIRD) and the non-diseased group (2) The vaccine effectiveness for each of the three disease groups (MS, IBD, CIRD), stratified by the categorized severity of received medication in regards to immunosuppressive effect. Both contrasts will be estimated as *intention-to-treat* and *per-protocol* effects. The target estimand is the average causal effect on the treated (ATT).	
Statistical analysis	Computing formal contrasts of simple Kaplan-Meier estimates.	Time-dependent 1:1 matching by days without replacement. Controls will be matched exactly by age in years, biological sex, place of residence, and disease status. The matched sample will be analyzed using a Cox model with additional adjustment for further confounders. G-computation based on this Cox model and a non-parametric estimate of the baseline hazard will be used to estimate the target estimand. Both *intention-to-treat* and *per-protocol* effects will be estimated by different censoring schemes as described in Section 2.6.3.
Sensitivity analysis	None.	The entire analysis will be repeated using *medically attended urinary tract infection* as a negative control outcome to assess the robustness of the confounder adjustment employed.
Reporting guideline	The results of the trial would be reported according to the *Consolidated Standards of Reporting Trials* (CONSORT) statement (78).	The results of the trial will be reported according to the *Reporting of Studies Conducted using Observational Routinely-Collected Health Data* (RECORD) statement (79).

a The 01.12.2018 is the first day from which the inactivated herpes zoster vaccine was recommended by STIKO (in german: “Ständige Impfkommission”) in Germany (29).

b Although information for two subsequent target trials (01.09.2019–31.05.2020 and 01.09.2020–31.05.2021) would be available, we choose not to try to emulate trials during this period because of the COVID-19 pandemic happening at the same time, which greatly impacted the spread and diagnosis of influenza.

Below we give a short first description of the considered target trials. More detailed information on the inclusion- and exclusion criteria as well as population definitions are given further below, because they are equal to the definitions used in the actual study. Due to the different nature of the considered vaccines, two different kinds of target trials will be described: one for the herpes zoster vaccination and one for each of the influenza vaccination trials.

The target trial for the herpes zoster vaccine effectiveness study starts at the first December of 2018, which is the date on which the vaccine became available in Germany. Each day from then on, people will be assessed for eligibility. If they meet the inclusion criteria, they are included in the study and are then randomized to either receive the vaccination (treatment group) or to receive nothing (control group). All individuals in the treatment group immediately receive the first dose of the vaccination. A second dose is administered after two to six months, in accordance to the current guidelines (29). All trial participants are followed up until the trial ends at the 31.12.2021. The primary outcome is then defined as the days until the occurrence of the main event of interest, where the start date is the date of trial inclusion for each individual.

The target trial for the influenza vaccine is very similar, but differs slightly due to the seasonality of the disease. Influenza viruses are constantly mutating, necessitating yearly changes of the influenza vaccines. It is therefore recommended to estimate influenza vaccine effectiveness on a seasonal basis instead (30). The target trial is therefore also defined on a seasonal basis, meaning that there is one target trial per influenza season that should be emulated. Recruitment would start each year on the first of September. People may then be recruited and followed up until the end of May of the next year (31.05.).

Both target trials are pragmatic, in the sense that no placebo control is used. We choose to formulate them this way because it is generally impossible to emulate placebo-controlled randomized controlled trials using only observational data (27). We further assume perfect adherence, so that every individual randomized to the treatment group actually receives the respective vaccination strategy and every individual in the control group does not. Our main interest is therefore in the *per-protocol* effect, although we will also estimate *intention-to-treat* effects in a sensitivity analysis, as described in detail in Section 2.6.3. Because we compare the vaccine effectiveness of patients with autoimmune diseases and other patients, but are unable to randomize the disease status itself, we are interested mainly in the *effect modification* of the disease status on the vaccine effectiveness (31). The result is a comparison of two population-based total causal treatment effects (32).

### 2.3 Study design and data source

Routinely collected claims data from a large German statutory health insurance (BARMER) will be used to carry out the study. BARMER is the second largest statutory health insurance in Germany with about 8.6 million insured individuals (status of 2024). BARMER claims data are available on application for research purposes in anonymized data sets, hosted in a scientific data warehouse [in German: Wissenschaftliches Data Warehouse (WDWH)] by BARMER. Within the project VAC-MAC (in german: VACcinierung von MS/Arthritis/Colitis-Patient:innen, DRKS-ID: DRKS00031559, registered 28.08.2023), we have access to data from 2013 to 2021. VAC-MAC is a project funded by the German Innovations Fund (Innovationsfonds, grant number 01VSF21044) with multiple goals related to assessing and improving the vaccination coverage of individuals with MS, IBD, or CIRD.

The target population of all target trials consists of all individuals insured by the BARMER who fulfill the following inclusion criteria. The subject:

1. Has been enrolled in the BARMER health insurance company for at least 2 years before inclusion into the study with interruptions of no more than 5% of the total time.
2. Is at least 18 years old at the start of observation.
3. Has a biological sex of either male or female.
4. Did not receive an organ transplantation in the last 2 years before inclusion into the study.
5. Did not have any form of cancer in the last 2 years before inclusion into the study.
6. Did not have an autoimmune-related disease other than MS, IBD, or CIRD in the last 2 years before inclusion into the study.

The start of the observation period/study is defined as the date on which an individual is included in the study during the trial emulation, as outlined in Section 2.6. In addition to these criteria, we also define one trial-specific inclusion criteria for each vaccine-effectiveness analysis. For the herpes zoster vaccine-effectiveness study, we require that the individual has not previously received a herpes zoster vaccination. Similarly, for the influenza vaccine-effectiveness trials, we require that the individual has not already received an influenza vaccination in the current influenza season.

A detailed list of all International Statistical Classification of Diseases and Related Health Problems (ICD)-10 Codes used to assess the inclusion criteria is given in the online supplement. We require two years of previous enrolment in the BARMER because we would otherwise be unable to obtain information on all relevant confounders as described in Section 2.5.4. In these 2 years of enrolment, small interruptions are allowed (up to 5% of the total time), as a compromise between the potential for misclassification that arises, because no information is recorded when a person is not insured at BARMER, and the available sample size. The studies are further constrained to contain only biological males and females due to small amounts and inconsistent coding of the “diverse” category in the BARMER data. People with cancer, previous organ transplantations and other autoimmune diseases are excluded due to the effect these conditions and/or their treatment have on the immune system. There are no other exclusion criteria in this study. Special populations, such as pregnant women and people with other chronic or life-threatening diseases are also eligible to be included in the study.

### 2.4 Study period

Although information between 2013 and 2021 is available, we will not use this entire duration as a study period for all of the target trial emulations. First, as described in Section 2.2, there will be one trial emulation per influenza season, with only the time during the respective season being included. Secondly, the first influenza vaccine effectiveness trial emulation will start with the influenza season of 2015/2016 and not with influenza season of 2013/2014, because we need two years of previous follow-up to determine eligibility of the individual and to measure the relevant confounders. Finally, although information for a fifth trial emulation in the influenza season of 2019/2020 would be available, we will not perform this analysis. The reason for this decision is that the COVID-19 pandemic started during this season and largely overshadowed the influenza season of this year. The likely large amounts of underreporting of influenza infections during the COVID-19 pandemic, combined with the actual effects of the pandemic and the measures taken to combat it (33), make a target trial emulation infeasible. Similarly, the target trial for the herpes zoster vaccine effectiveness analysis only starts on the 01.12.2018, because the inactivated herpes zoster vaccine was not available in Germany and not recommended by the STIKO (in German: “Ständige Impfkommission”) before this date (29).

### 2.5 Measures

#### 2.5.1 Disease definitions

We will consider three main types of autoimmune disease groups: MS, CIRD, and IBD. To determine whether an individual has developed one or multiple of the diseases contained in each group at certain points in time using the BARMER data, we developed new algorithms based on previous research. For MS all existing subtypes of the disease are considered. Our algorithm to identify MS is loosely based on the algorithm developed by Culpepper et al. (34). We further define an individual to have CIRD only when that individual either has *rheumatoid arthritis, axial spondyloarthritis* or *systemic lupus erythematosus*. Those three diseases are each identified using different algorithms, based on previous work (35–37). Similarly, we define IBD to be present if the individual has *Crohn's disease* or *colitis ulcerosa*, which are also identified separately using algorithms based on previous research (38, 39). The exact definitions used for each disease and sub-disease are given in the online supplement of this article.

Through the course of the VAC-MAC project, these definitions have already been applied to the BARMER data. We were able to identify approximately 50,000 patients with MS, 300,000 patients with CIRD and 80,000 patients with IBD between the 01.01.2013 and the 31.12.2021. Additionally, approximately 9,000 of these patients had more than one of the three considered disease categories. Although this constitutes a “look into the data” before this study protocol was published, this procedure is in accordance with recommendations by Wang and Schneeweiss (40), who state that performing data checks before study registration actually increase the validity of the study.

#### 2.5.2 Treatments

The “treatments” in this study are the two different types of vaccines of interest. We will not differentiate between different subtypes of vaccines in the influenza vaccine effectiveness trials. Any vaccine against influenza will be considered an “influenza vaccine”, regardless of its' underlying mechanism and irrespective of which company created the vaccine. In the herpes zoster trial we will only consider the inactivated recombinant zoster vaccine (Shringrix^®^) (41). In Germany, vaccinations are usually provided by physicians in primary care. Physicians receive a reimbursement for each vaccination from the statutory health insurance of the individual being vaccinated. To this end, a standardized reimbursement code [German: Gebührenordnungsposition (GOP)] is used and transferred to the insurance as part of a reimbursement claim. We will use these codes to identify vaccinations in our study population, using both codes applying uniformly for all regions in Germany and codes only used for specific regions and time periods. The reimbursement codes used are listed in the online supplement.

There is, however, one special case that needs to be mentioned. Whenever individuals get vaccinated through a company physician (in German: Betriebsärzte), the reimbursement code is not transferred to the insurance company of the individual. The reimbursement is instead taken care of by the companies themselves. Additionally, a few pharmacies and hospitals also vaccinate individuals without sending reimbursement codes to the insurance company (42). Because these vaccinations never show up in the individuals data of the health insurance, the vaccination status of these individuals will be misclassified in the analysis. We expect this issue to have a minor impact, because the majority of vaccinations are performed by primary care physicians in Germany. There is currently no official data available to quantify the proportion of vaccinations performed in these different settings. Due to the complex nature of the proposed analysis strategy, as described in Section 2.6, it is also not possible to apply standard methods of quantitative bias analysis to quantify the effects of this potential bias (43). To still get a rough estimate of the potential impact of this misclassification, we will repeat the main analysis after removing 10% of all vaccination dates from the available data in a sensitivity analysis.

#### 2.5.3 Outcomes

Different outcomes will be used for each of the considered vaccine effectiveness trials. For the emulation of the proposed target trials, the date on which the respective outcomes occurred is required. Our aim is therefore to identify this date of occurrence given the available insurance claims data. In the German system, ICD-10 codes that were used in the inpatient setting are directly associated with an exact date. Outpatient ICD-10 diagnosis, however, are only associated with a time interval which may be up to 91 days (one quarter) long, depending on the patient. In some cases, the corresponding date of the diagnosis must therefore be identified using associated treatments or doctors certificates on the incapacity to work, for which exact dates are available. The concrete strategy used to identify the dates for outpatient diagnosis of each defined outcome and associated sensitivity analyses are described in the online supplement.

The outcome for the herpes zoster vaccine effectiveness analysis is a *medically attended herpes zoster infection*. This outcome will be identified from the available data through the use of ICD-10 code **B02** (inpatient or outpatient diagnosis), in conjunction with a prescription for an antiviral drug (acyclovir, valacyclovir, famciclovir, or brivudin). This is similar to the definition proposed by Zerbo et al. (44), which was estimated to have a positive predictive value of 96.7% by the same authors. Other studies additionally employed information about positive laboratory tests (45), which is not possible in this study due to a lack of available data.

For the influenza vaccine trials we will use *medically attended influenza* as the primary outcome, as recommenced in the literature (30, 46). We define a medically attended influenza as an influenza infection that results in any type of formal contact between the patient and a health care professional. In particular, we define a medically attended influenza infection as having occurred when either one of the ICD-Codes **J09**, **J10**, or **J11** has been used as a secured outpatient diagnosis or in an inpatient main or secondary diagnosis. The medically attended influenza is considered to be *severe* if it is associated with a hospital admission due to pneumonia or death. We will use the severe medically attended influenza as a secondary outcome.

#### 2.5.4 Confounders

**Figure 1:**
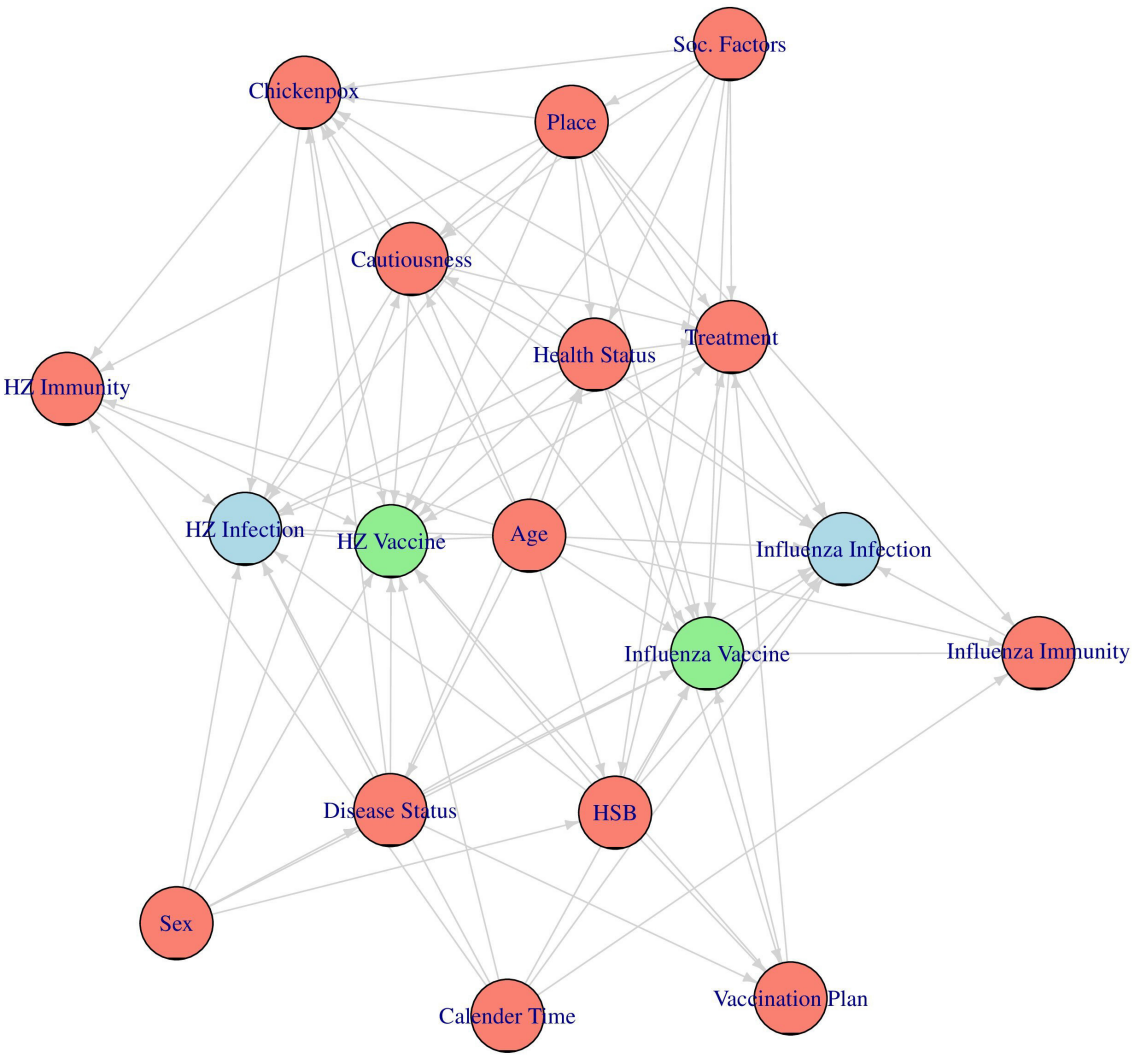
The assumed causal directed acyclic graph (DAG). The two vaccines of interest are drawn in green, the two main outcomes are drawn in blue and the considered confounders are drawn in red.

Since this is an observational study and the vaccinations were not randomly assigned, confounding needs to be adjusted for in order to correctly emulate the target trials. To identify a sufficient adjustment set of confounders for each analysis we first built a directed acyclic graph (DAG), encoding our assumptions about the causal structure of the data (47). In this graph each node corresponds to one variable or concept while the directed arrows between them denote directed causal effects. The absence of an arrow between two nodes is equal to the assumption that there is no direct causal effect of one node on the other. We built this DAG primarily based on a previously suggested DAG by Stuurman et al. (48) and Remschmidt et al. (49), including additional information extracted from other relevant studies (30, 50–52) and through expert discussion. The resulting DAG is shown in Figure 1.

To make the DAG more comprehensible, we excluded the time dimension from it, even though all of the considered nodes are actually time-dependent. For the same reason we also grouped together all factors that are hypothesized to have the same causes under one concept each, as was done in Stuurman et al. (48). An explanation of each considered variable and concept, as well as a list of indicators used to represent them in the actual analysis (if necessary), is given in Table 2. The variables corresponding to the respective vaccines [*Influenza Vaccine* and *HZ Vaccine* (herpes zoster vaccine)] and the variables corresponding to the associated main outcomes [*Influenza Infection* (medically attended influenza) and *HZ Infection* (medically attended herpes zoster infection)] are not listed in Table 2, because they have been described in detail earlier.

**Table 2:** A description of the variables or concepts and a list of corresponding indicators that could and/or will be used to measure them.

**Description**	**Indicator(s)**
**Age**	
The age of the patient in years.	The year of birth is used to obtain the age in years.
**Sex**	
The biological sex of the patient (male/female).	This variable is directly recorded in the BARMER database.
**Calender time**	
The date of the inclusion into the study.	This variable is also directly recorded in the BARMER database.
**Place (Place of residence)**	
The place of residence of the individual.	The first three digits of the postal code of an individual or the associated federal state will be used as a rough proxy for the place of residence.
**Soc. factors (Socioeconomic factors)**	
Socioeconomic status and ethnicity of an individual.	None available.
**HSB (Healthcare-seeking behavior)**	
Healthcare seeking behavior, defined as the individuals propensity to seek medical care when ill or perceived ill.	Although not directly observable, several indicators of HSB can be identified from the data (80). We will consider: (1) The number of primary care physician visits in the year before study entry (48). (2) The total number of visits to a physician in the year before study entry. (3) Whether the individual has received a pneumococcal vaccination in the 2 years before study entry, similar to recommendations by World Health Organization (30). (4) Whether the individual has participated in a voluntary preventive medical check-up as coded by the “Einheitliche Bewertungsmaßstab Ziffer” (EBM) number 01732 in the last 2 years.
**Cautiousness**	
The degree of cautiousness displayed by an individuals social contact patterns and general precautionary social behavior.	Stuurman et al. (48) mention the number of people living in the individuals household and the presence of a child in the individuals household as indicators for this variable. This information is, however, not available in the BARMER database.
**Health status**	
The general health status of an individual. This includes both physical and psychological wellbeing.	Two indicators will be used as proxies for this variable. We will consider: (1) A modified version of the Charlson comorbidity index (81) that does not include the main diseases of interest in this study, calculated using ICD codes from the last year. (2) The number of inpatient hospital stays in the last 2 years. (3) The current official level of care.
**Treatment**	
The kind of medical treatment an individual has received in the past and is currently receiving.	A variable that classifies the medical treatment an individual has received in the last year into different categories of severity in regards to immunosuppressive effect. More details are given in the online supplement.
**Disease status**	
Whether the individual belongs to one or more of the three disease groups considered in this study.	The exact definition of each disease is given in Section 2.5.1.
**Influenza immunity**	
Whether the individual already has acquired some immunity against influenza through previous influenza vaccinations or a previous influenza infection.	We will consider: (1) Whether the individual has received an influenza vaccination in the previous year. (2) Whether the individual has experienced an influenza infection in the previous year.
**HZ immunity (Herpes Zoster immunity)**	
Whether the individual already has acquired some immunity against herpes zoster through a previous infection.	An indicator of whether the individual has experienced a medically attended herpes zoster infection in the previous six month will be used.
**Chickenpox**	
Whether the individual has ever been infected with chickenpox, also known as varicella.	This will be measured using the corresponding ICD-10 code **B01**. Because only information between 2013 and 2021 is available and chickenpox usually occur during childhood, most of the actually occurred infections will be missed.
**Vaccination plan**	
For individuals with MS, IBD, or CIRD it is not unusual that the vaccination is planned in advance by the physician and aligned with changes in medication that may have immunosuppressive effects. For example, the physician may decide that the individual should be vaccinated next week. To allow the patients immune system to make a proper response to the vaccination, a new prescription of immunosuppressive medication is then delayed for a few weeks. This variable states whether such a plan was made or not.	An indicator of whether a person receives a medication change shortly after vaccination may be created. However, this type of indicator breaks the time-line, since information from the future would be used. We will perform an additional sensitivity analysis to determine the effect of using this indicator as an adjustment variable.

Using this DAG and the *backdoor criterion* (47), we then identified separate sets of factors that we need to adjust for in each vaccine effectiveness analysis (53). These sufficient adjustment sets are very similar. In both of the two considered analyses, we will adjust for *Age, Sex, Place, Calender Time, Cautiousness, Disease Status, Healthcare-Seeking Behavior* (HSB), *Treatment, Health Status*, and *Vaccination Plan*. The only differences are that we additionally have to adjust for *Chicken Pox* and *Herpes Zoster Immunity* in the herpes zoster vaccine effectiveness analysis and that we also have to adjust for *Influenza Immunity* in the influenza vaccine effectiveness analysis.

Note that, given our assumptions, there is no need to adjust for socioeconomic status in either analysis, because we hypothesize that the effect of this variable on each outcome is only indirect and is thus already adjusted for by including the other variables. It is also not strictly necessary to adjust for sex in the influenza vaccine effectiveness analysis for the same reason. We choose to include sex anyway, because it can be seen as a proxy for other relevant confounders such as cautiousness that we may not be able to fully adjust for given the mentioned indicators (48).

For most of the relevant confounders identified through the DAG, one or multiple indicators can be extracted from the available BARMER data, as described above. However, this is not the case for all confounders. For example, we were unable to identify valid indicators for the *Cautiousness* of an individual. We are thus unable to adjust for all confounders, potentially resulting in a violation of the *conditional exchangeability* assumption (e.g., that the groups are exchangeable given the observed confounders) (32). Although social contact behavior is a crucial factor in infection transmission and thus an important predictor for the outcomes in our study, its role as a predictor for vaccination decisions is less clear. A study by Ibuka et al. (54) indicates that people with less cautious contact behavior only have a slightly higher chance of getting vaccinated. We therefore expect the bias introduced by this unmeasured confounder to not be substantial.

An additional issue in the adjustment is that not all considered confounders are measured perfectly. In particular, the influenza and herpes zoster immunity at baseline will likely be underestimated, because not all individuals who experienced an infection visited a medical professional because of it. Hence, not all infections are included in the data, resulting in some individuals appearing as not having been infected, despite having been infected. Furthermore, because we only have access to the data between 2013 and 2021, we will likely underestimate the true extent of the existing herpes zoster immunity as well, because most chickenpox infections happen during childhood and we did not measure childhood vaccinations. These measurement errors may result in additional residual confounding (43). Sensitivity analysis will be performed to further quantify this potential source of bias, as described in Section 2.6.6.

### 2.6 Data analysis plan

The main analyses will be conducted using a combination of time-dependent matching (55, 56) and a multivariable Cox proportional hazards regression model (57). Similar approaches have been used to study the effectiveness of COVID-19 vaccines (28) and are generally quite popular when analyzing the effect of a time-dependent variable on a time-to-event outcome (58). Below we first describe how the matching will be performed. Afterwards a detailed explanation of the planned analysis of the matched data is given, as well as a description of how possible intercurrent events are handled, a discussion of the required assumptions and the planned sensitivity analysis.

#### 2.6.1 Matching method

Due to the different target trials per vaccine of interest, the planned matching and analysis strategy also differs slightly. To analyze the effectiveness of the herpes zoster vaccine, a single matching process will be used. For the influenza vaccine effectiveness trials we will use the same procedure, with the only difference being that it will be performed separately for each influenza season. The matching will be carried out as follows.

For the previously defined start date of the respective trial, the eligibility of all individuals contained in the BARMER database will be determined using the above mentioned inclusion criteria. Eligibility will be checked using only the information available at that time. Every individual who was vaccinated with the vaccine of interest on this date is subsequently included in the dataset, in the treatment group. For every individual included in this way, we then include *k* further individuals who fulfill the inclusion criteria, but have not been vaccinated yet, in the control group. These controls will be matched exactly by age in years, gender, place of residence and disease group. If more than *k* potential controls are identified for a case, *k* controls will be picked at random from these. We will try to include 2 controls per case first (*k* = 2), but will lower this number to 1 if the number of available matches is insufficient. The date of inclusion equals “time zero” (27) for all individuals. Information about all relevant confounders is recorded as available on this date, corresponding to baseline information. This procedure is repeated on all subsequent days, until the emulated trial is over.

Controls will be matched without replacement, meaning that individual *i* who was used as a control at one point in time *t* cannot serve as control more than once at the same point in time and can also not be used as control again at later points in time. However, people who have been used as controls will still be included in the trial later, if they get vaccinated. They will then be included in the treatment group, again with the vaccination date corresponding to the trial inclusion date. Information about the different defined outcomes are extracted from the BARMER data for each individual. This matching procedure results in datasets that are similar to what we would obtain if we actually had performed the respective target trials. The main advantage of this method is that it directly matches patients by inclusion-date, giving us a clearly defined “time zero” (27). Because controls are choosen to be similar to vaccinated individuals, this matching methods targets the *average treatment effect on the treated* (ATT) (56).

If no suitable control can be identified for a vaccinated individual at the time of vaccination, the individual is not included in the analysis. Because of the large amount of data, the long follow-up time and the fact that only a relatively small proportion of individuals gets vaccinated, we believe that only a small proportion of individuals will be excluded. If the proportion of vaccinated individuals with no matched controls exceeds 10%, we will adjust the choice of matching variables to lower this proportion. This will be done by either widening the age groups or not matching by sex. In theory, methods other than exact matching, such as generalized full matching (59) would offer a viable alternative in such cases. It would also allow matching on more confounders, which would be preferable. We unfortunately cannot use such methods, due to the high computational complexity of our approach and the limited available technical infrastructure.

#### 2.6.2 Analysis method

The matching process described above reduces the time-dependent vaccination variable to a time-fixed binary variable, allowing the use of well known analysis methods. We will first describe the matched data using descriptive statistics, stratified by treatment group and disease status. Means and standard deviations will be calculated for normally distributed continuous variables, median and ranges will be used for non-normally distributed continuous variables and counts and percentages will be calculated for categorical and binary variables. Appropriate statistical tests, such as *t*-tests and chi-squared tests, will be used to formally compare the distribution of these variables at baseline. Histograms and boxplots, stratified by both treatment groups and disease groups will also be drawn. In addition, the number of vaccinated individuals, matched controls and number of potential controls evolving over time will be graphically displayed as recommended by Thomas et al. (56).

For the estimation of the vaccine-effectiveness, we will employ standard time-to-event analysis methods, such as Kaplan-Meier curves and Cox models. No complex marginal structural models or similar strategies are required, because only information before inclusion into the trial is used for adjustment, making it impossible for *treatment-confounder* feedback (32) to occur.

Because we only match by age in years, gender, place of residence and disease status, we still have to adjust for the other confounders mentioned earlier to obtain unbiased estimates of the average causal effect we are interested in. A Cox model using the time to the respective outcome of interest as dependent variable and the treatment variable, the disease status and all relevant confounders (as observed at trial inclusion) as independent variables will be used to perform this adjustment. Since we are interested in the effect-modification of the vaccine effectiveness by disease status, we will also include an interaction term of these two variables in the model. This model, in conjunction with an estimate of the baseline hazard function (60), will then also be used to estimate confounder-adjusted survival curves using g-computation (61). These curves will then be used to derive time-dependent relative risk estimates, which will in turn be used to derive the vaccine effectiveness as (62):


(1) $$\begin{array}{l} \theta \left(t\right)=1-RR\left(t\right), \end{array}$$

by disease status, where *t* denotes the point in time and *RR*(*t*) is the relative risk of experiencing the respective outcome at time *t* in the vaccinated vs. unvaccinated group. Formal contrasts of the disease specific vaccine effectiveness estimates will then also be calculated. Cluster-robust standard errors will be used in the Cox model to adjust for potential dependencies introduced by matching (63). Non-parametric bootstrapping will be used to derive confidence intervals for the vaccine effectiveness and their formal comparisons. Although Abadie and Imbens (64) demonstrated analytically that bootstrapping is not a valid strategy to estimate standard errors in matched samples, it has been shown to work well in simulation studies (65). Due to these simulation results and a lack of alternatives we will therefore still rely on this method.

A very similar analysis strategy will be used to assess the moderating effect of the prior use of immunosuppressive medication on the vaccine effectiveness. In this analysis, only individuals with MS, IBD, or CIRD will be included. The entire analysis described above is then repeated, stratified by disease group and the severity of the received immunosuppresive medication (if any). Additionally, g-computation will be used to estimate the age-specific vaccine-effectiveness in each trial. To obtain a single estimate for the influenza vaccine, we will also use standard meta-analytic approaches to combine the estimates obtained from each of the four emulated influenza trials.

The proportional hazards assumption will be checked using Schoenfeld residual plots (66). If the assumption is deemed violated, we will try different strategies, such as inclusion of time-dependent hazard-ratios and variable transformations, to alleviate this issue. If these methods are unsuccessful, other models such as the additive hazards model (67) with a similar specification will be employed instead. In addition, we will perform a sensitivity analysis in which we will use a weighted Kaplan-Meier estimator instead of g-computation (68), where the weights will be estimated using entropy balancing (69).

#### 2.6.3 Intercurrent events

Four possible types of events that may happen to individuals after inclusion into the study need to be accounted for. These possibly events are:

1. Death.
2. Loss to follow-up.
3. Individuals receiving another unplanned vaccination after the first dose (e.g., non-adherence to the “assigned” treatment status).
4. Individuals *not* receiving a planned second vaccination (only applicable to the herpes zoster vaccine effectiveness study).

Of course, death may occur at any time for any number of reasons, while loss to follow-up may only occur when individuals terminate their enrollment in the BARMER health insurance company. The third type of intercurrent event requires more considerations. In practice, individuals may receive another vaccination after already having received the recommended amount of doses. Additionally, because only information at the time of inclusion is used when performing the matching, it is possible that individuals which were selected as controls get vaccinated themselves during follow-up. Lastly, because the matching is performed on the date of first vaccination, it is possible that individuals do not receive a planned second vaccination as recommenced for herpes zoster vaccines.

In all analyses, individuals will be considered right-censored at their time of loss to follow-up or death (whichever occurs first). In the primary *per-protocol* analysis, the event time of individuals will also be censored at the time at which they receive a vaccination that does not correspond to the planned treatment strategy, or at the time at which they should have received a second vaccination but did not do so. This way, only time during which the individuals adhered to their assigned treatment is considered in the analysis. This strategy likely results in covariate-dependent censoring, which will be accounted for by also censoring the matched pair of the individual at the same time (56). In the secondary *intention-to-treat* analysis, the subsequent vaccinations are ignored instead, which results in all potential effects of non-adherence to the assigned treatment to be included in the analysis.

#### 2.6.4 Missing data

Most of the variables required for the proposed analysis, with the exception of some demographic variables such as age, sex and place of residence, are based on the presence of ICD-10 codes or similar codes in the BARMER data. As such they are not subject to have known missing data. For example, if an individual does not have any ICD-10 codes related to an influenza infection, the individual will be coded as not having experienced a medically attended influenza. Although it is possible that the individual *did* experience a medically attended influenza that was not properly recorded in the claims data, these “missing” data entries will not be known to us. Nevertheless, because all such variables we are interested in are relevant for proper accounting, we believe that this is a very minor issue. Preliminary analysis through the course of the VAC-MAC project additionally indicate that the amount of missing data in demographic variables is negligible. Accordingly, individuals with missing data will simply be removed from the analysis without the use of imputation based or similar strategies (70).

#### 2.6.5 Assumptions

Because the aim of this study is to estimate causal effects, we have to make the four standard causal identifiability assumptions (conditional exchangeability, positivity, counterfactual consistency, and no interference). These are described in detail elsewhere (32). As mentioned earlier, the conditional exchangeability assumption may be violated here, which means there may be some residual confounding left after adjustment. Additionally, the no interference assumption, which states that the vaccination status of one individual does not have an effect on the outcome of another individual, is also probably violated, as is usually the case in vaccine effectiveness studies (71). This, however, has nothing to do with the observational nature of this study. The same violation would occur if the randomized target trial was actually carried out, with no commonly accepted solution to this problem having been proposed in the literature (71, 72). The results of this study will therefore have to be interpreted cautiously.

#### 2.6.6 Negative control outcome analysis

To quantify the amount of residual confounding, a negative control outcome analysis will be performed (73). In this strategy, the analysis is repeated with suitable negative control outcomes, which are variables that ideally have the same causes as the actual outcome of interest, but are not directly caused by the exposure of interest. We will use *medically attended urinary tract infection* as a negative control outcome for all considered analyses. In particular, we will use inpatient and outpatient diagnoses (ICD-10 code N39.0) with an associated prescription for a relevant antibiotic to identify the infections (see online supplement). This outcome has been used in other vaccine effectiveness studies for influenza- (73, 74), herpes zoster- (75), and pneumococcal vaccines (76). These studies additionally used other negative control outcomes, such as hip fractures and fractures of the hand or wrist. We choose not to use these outcomes here, due to their potentially large association with corticosteroid medication (77), which we can only partially adjust for in our analysis through the category of received medications. Our strategy to identify the exact dates of outpatient diagnosis is again described in the online supplement.

## 3 Discussion

The proposed study has multiple strengths. One of the biggest strengths is its large sample size. With approximately 50,000 MS patients, 300,000 CIRD patients, 80,000 IBD patients and potentially millions of control patients, this study is, to the best of our knowledge, the largest of its kind. Because it is based on data from a German health insurance provider, it also contains information on the medications prescribed to each patient, as well as their acquired infections and comorbidities. This large sample size makes a detailed disease specific population based comparison of the vaccine effectiveness with appropriate controls possible. The available information also makes adjustment for many of the relevant confounders feasible. The very limited amount of inclusion criteria additionally facilitates the generalizability of the obtained results.

A limitation of this study is the potential of unmeasured or unknown confounding. As discussed earlier, we are not able to adjust for all confounders that were identified by our assumptions about the causal data generation process. For example, it is impossible to measure social contact behavior from the available data. It is also possible, if not likely, that the DAG we specified is partially wrong or fails to include other, currently unknown, confounders. This is a frequent problem in observational studies, although it is not always acknowledged (32). We will attempt to quantify the impact of this potential source of bias through pre-planned sensitivity analyses.

A further limitation is the potential of misclassification of the vaccination status due to individuals getting vaccinated through their company physicians, or in rarer cases in hospitals or pharmacies. Because some individuals will be misclassified as “unvaccinated,” it is possible that the estimates of the vaccine effectiveness will be biased downwards. However, we expect this to be a minor issue, because most individuals in Germany get vaccinated through their primary care physician. Under the assumption that the misclassification rate does not differ between the considered disease groups, we would also not expect bias of the contrast between the vaccine effectiveness estimates.

Additionally, it is important to note that the BARMER data is not a probability based sample of the general German population. It is possible that there are substantial differences between the general German population and people enrolled in the BARMER, which theoretically makes the generalization of the results of this study to the general German population difficult. Nevertheless, due to its large size and nation-wide adoption, we believe that this is a minor problem.

## VAC-MAC Consortium

Kerstin Hellwig and Thomas Grüter, Department of Neurology, St. Josef Hospital, Ruhr University Bochum, Bochum, Germany; Nina Timmesfeld, Marianne Tokic, Jale Basten, Robin Denz, and Katharina Meiszl, Department of Medical Informatics, Biometry and Epidemiology, Ruhr University Bochum, Bochum, Germany; Ingo Meyer, Katja Blaschke, Heike van de Sand, and Carolin Heinen, PMV Research Group, Medical Faculty and University Hospital Cologne, University of Cologne, Cologne, Germany; Stephanie Stock, Dusan Simic, and Arim Shukri, Institute of Health Economics and Clinical Epidemiology (IGKE), Faculty of Medicine and University Hospital Cologne, University of Cologne, Cologne, Germany; Uta Kiltz and Maria Zacharopoulou, Rheumazentrum Ruhrgebiet, Ruhr University Bochum, Bochum, Germany; Horst Christian Vollmar, Ina Otte, Chantal Giehl, Romy Lauer, and Anastasia Suslow, Ruhr University Bochum, Institute of General Practice and Family Medicine (AM RUB), Medical Faculty, Bochum, Germany; Andreas Stallmach and Anika Franz, Department of Internal Medicine IV (Gastroenterology, Hepatology and Infectious Diseases), Jena University Hospital, Jena, Germany; Ursula Marschall, Joachim Saam, and Catharina Schumacher, Department Medicine and Health Services Research, BARMER Institute for Health System Research, Wuppertal, Germany; Katja Matthias, Daniel Salas, Jill-Marie Fix, Sarah Peitz, Philipp Lutz, and Iris Hermanski, TAKEPART Media + Science GmbH.
