# Supplementary material for: Comparing the effectiveness of different vaccines in individuals with and without autoimmune diseases: a study protocol for a target trial emulation
Source: Front Public Health. 2025 May 26;13:1583434. doi: 10.3389/fpubh.2025.1583434 (PMC12146351; doi:10.3389/fpubh.2025.1583434)
Supplement: Supplementary file 1 [file Data_Sheet_1.pdf]

Supplementary Material

1 ELIGIBILITY CRITERIA DEFINITION

For all analyses proposed in the study protocol, we aim to exclude individuals with cancer, a previous organ transplantation or autoimmune diseases that are not multiple scelrosis (MS), chronic inflammatory bowel disease (IBD) or chronic inflammatory rheumatic disease (CIRD). In table S1 the relevant International Statistical Classification of Diseases and Related Health Problems (ICD)-10 codes used to identify these criteria are shown. Individuals who received at least one of these codes in the last two years are not eligible to enter the study at this time. In theory it would be more appropriate to formulate more precise definitions of the three eligibility criteria, as we did with the three diseases of interest (MS, IBD, CIRD). Due to the immense amount of work the creation and application of these definitions would require, this is unfortunately not feasible in the present study. However, we think that this very broad definition is still suitable for our purpose, which is to exclude individuals without MS, IBD or CIRD who have a strongly impacted autoimmune system.

Since these eligibility criteria are defined using ICD-10 codes, we cannot easily extract exact dates for each diagnosis made due to the structure of the available data, as described in the main text and below in the next section. To obtain these dates for each ICD-10 code in the outpatient setting would require formulating a meaningful identification strategy separately for each code, which is unfortunately not feasible due to time constraints. Instead, the presence of these ICD-10 codes will only be checked on a quarterly basis. If patients had either of these diagnosis in the last 4 quarters (not including the current quarter) they will not be included in the respective emulated study.

Table S1. ICD-10 Codes used to define the eligibility criteria

Description	ICD-10 Codes
Cancer	C00-C97
Organ Transplantation	Z94
Alopecia areata	L63
Autoimmune enteropathy	D89
Autoimmune hepatitis	K75
Polyendocrine autoimmune diseases	E31
Bullous pemphigoid	L12.0
Chronic type A gastritis	K29
Eosinophilic granulomatosis with polyangiitis	M30.1
Chronic inflammatory demyelinating polyneuropathy	G61.8
Dermatomyositis	M33.0, M33.1
Dermatitis herpetiformis Duhring	L13.0
Endocrine orbitopathy	H06.2*
Epidermolysis bullosa	Q81
Glomerulonephritis	N00.-N01., N03.-N05., N08.-*
Goodpasture-Syndrome	M31.0
Granulomatosis with polyangiitis	M31.3
Guillain-Barré-Syndrom	G61.0
Hashimoto's thyroiditis	E06.3
Idiopathic thrombocytopenic purpura	D96.3
Lichen sclerosus	L90.0, N48.0, N90.4
Lichen ruber planus / Lichen planus mucosae	L43
Linear IgA dermatosis	L13.8
Lupus erythematosus	L93.0, L93.1, L93.2
Microscopic polyangiitis	M31.7
Morbus Basedow	E05.0
Morbus Behçet	M35.2
Myasthenia gravis	G70, G70.0
Narcolepsy	G47.4
Parkinson's disease	G20, G21, G22*
Pemphigus foliaceus	L10.2
Pemphigus seborrhoicus / Pemphigus vulgaris	L10.0
Polychondritis	M94
Polymyalgia rheumatica	M35.3, M31.5
Polymyositis	M33.2
Psoriasis	L40
Pyoderma gangraenosum	L88
Rheumatic fever	I00, I01, I01.0, I01.1, I01.2, I01.8, I01.9, I02.0, I02.9
Giant cell arteritis	M31.5, M31.6
SAPHO-Syndrome	M86.3(0-9)
Sarcoidosis	D86.0, D86.1, D86.2, D86.3, D86.8, D86.9
Sjögren-Syndrome	M35.0
Scleroderma	M34
Stiff-Man-Syndrome	G25.8
Sympathetic ophthalmia	H44.1
Purpura Schönlein-Henoch	D96.0
Vitiligo	L80
Coeliac disease	K90.0

2 OUTCOME DEFINITIONS

As discussed in the main text, our planned analysis strategy relies on date-specific information on the occurence of each outcome. Those dates are only directly available for ICD-10 codes coded in an inpatient setting. For outpatient diagnosis, special techniques have to be used to identify this date. The following sections describes our strategies for each of the three outcomes (herpes zoster infection, medically attended influenza and severe medically attended influenza) and the negative control outcome (urinary tract infection). It also describes some checks that will be performed to ensure that our definitions are meaningful.

Some preliminary information is needed before we can describe the strategies. In the German system, an *outpatient case* is defined on a quarterly basis. Whenever the first billable event in the outpatient setting happens for a patient during a quarter, the case starts. The case ends with the date of the last billable event in the outpatient setting during the same quarter. Regardless of the reason for the billing (visits to different medical professionals for different reasons), all outpatient diagnosis made for a particular patient in this quarter are only associated with this particular patient-specific case. If a patient experiences no contacts with the health care system in the outpatient setting, which are relevant for accounting, except the one with the diagnosis of interest, that day may be used directly as the date of diagnosis. However, if, for example, the patient went to a medical professional for a health check-up 30 days earlier in the same quarter, the diagnosis is only known to be in a 31 day window. Usually, this window can be narrowed down to a short time interval using additional information on given treatments and other codings, as described below.

2.1 Herpes Zoster Infection

Identifying the specific date of occurence of a herpes zoster infection will be done separately for inpatient and outpatient diagnosis. If the ICD-10 code B02 was coded in an inpatient setting the date of admission will be used, which is available directly in the data. If the diagnosis was made in an outpatient setting, the following strategy is used instead:

1. First, we try to identify the date through prescribed relevant medication. The list of all Anatomical Therapeutic Chemical Classification (ATC) System codes that are considered relevant medication for treating herpes zoster infections is shown in table S2. If such medication was prescribed to the patient, the prescription date of this medication is used as date of disease onset. If multiple such medicatons were prescribed to the patient during the case, the date of the first prescription will be used.
2. If no such medication could be identified in the relevant quarter, we consider the herpes zoster infection to not be present for this individual.

As a sensitivity analysis, we will also consider the following, slightly different approach, in which all ICD-10 codes are considered as true herpes zoster infections:

1. First, we try to identify the date through prescribed relevant medication. The list of all ATC codes that are considered relevant medication for treating herpes zoster infections is shown in table S2. If such medication was prescribed to the patient, the prescription date of this medication is used as date of disease onset. If multiple such medications were prescribed to the patient during the case, the date of the first prescription will be used.
2. If no such medication could be identified in the relevant quarter but a doctors certificate on the incapacity to work exists in this period with the corresponding same ICD-10 code, the date of the creation of this certificate is used as a disease onset date. If multiple such certificates can be found during the same case, the earliest date will be used.

3. If neither medication nor a doctors certificate on the incapacity to work exists in the same quarter as the ICD-10 diagnosis, but the current case duration associated with the diagnosis is at maximum seven days in length, the first day of the current case duration is used.
4. If no specific date could be identified in steps (1), (2) or (3), the mid-point of the case duration is used as the date of occurence.

For outpatient diagnosis, we will report the number and proportion of infections identified using points (1), (2), (3) and (4) as outlined above. In two additional sensitivity analyses we will set all dates of occurence identified in step (4) to the first and last date of the respective case duration and repeat the entire analysis using this modified outcome variable.

Table S2. ATC Codes used to define the eligibility criteria

Description	ATC Codes
Aciclovir	J05AB01, S01AD03
Famciclovir	J05AB09, S01AD07
Valaciclovir	J05AB11
Brivudin	J05AB15

2.2 Medically Attended Influenza

Identifying the specific date of occurence of a influenza infection will again be done separately for inpatient and outpatient diagnosis. If the ICD-10 codes **J09**, **J10** or **J11** were coded in an inpatient setting the date of admission will be used, which is available directly in the data. If the diagnosis was made in an outpatient setting, the following strategy is used instead:

1. First, if the duration of the current case is at maximum seven days long, the date when that case began is used as the infection date.
2. If the case duration exceeds seven days, but a doctors certificate on the incapacity to work is found in the same quarter as the relevant ICD-10 code, with that same ICD-10 code on it, the date of the creation of this certificate is used as a diseases onset date. If multiple such certificates can be found during the same case, the earliest date will be used.
3. If the case duration exceeds seven days and no doctors certificate on the incapacity to work exists in the relevant quarter, we try to identify the date through prescribed relevant medication. The list of all ATC codes that are considered relevant medication for treating influenza infections is shown in table S3. If such medication was prescribed to the patient by a medical professional of the same clinic in which the ICD-10 diagnosis was made (as identified by the facility number; in german: Betriebsstättennummer, abbr.: BSNR), the prescription date of this medication is used as date of disease onset. If multiple such medicatons were prescribed to the patient during the case, the date of the first prescription will be used.
4. If no specific date could be identified in steps (1), (2) or (3), the mid-point of the case duration is used as the date of occurence.

Table S3. ATC Codes used to specify the date of influenza infection

Description	ATC Codes
systemic antibiotics	J01
neuraminidase inhibitors	J05AH

For outpatient diagnosis, we will report the number and proportion of infections identified using points (1), (2), (3) and (4) as outlined above. In two additional sensitivity analyses we will set all dates of occurence identified in step (4) to the first and last date of the respective case duration and repeat the entire analysis using this modified outcome variable.

2.3 Severe Medically Attended Influenza

We define a severe medically attended influenza as:

1. any influenza diagnosis (ICD-10 codes **J09**, **J10** or **J11**) made in the inpatient setting (for which exact dates are available)

OR

2. any influenza diagnosis (inpatient or outpatient setting) with a subsequent death on the determined date of infection or within 14 days of this date

OR

3. any influenza diagnosis (inpatient or outpatient setting) with a subsequent hospitalisation due to pneumonia (ICD-10 code **J13**) on the determined date of infection or within 14 days of this date.

Exact dates for the death of individuals are available. This definition may be considered conservative, because the death is not necessarily due to the influenza. The cause of death is unfortunately not included in the available data and can thus not be used to validate this definition. We do, however, think that this is a minor issue. Because the date of the influenza infection in the outpatient setting is sometimes only known to lie in an interval (see above section), we will again perform two additional sensitivity analyses as described in the above section.

2.4 Medically Attended Urinary Tract Infection

To identify the date of disease onset for a urinary tract infection, the same strategy that was used to identify the dates for herpes zoster will be used. This includes the sensitivity analysis. The only difference is that it will be based on ICD-10 code N39.0 instead, and different relevant medication is used as listed in table S4. Other studies have used similar strategies to identify urinary tract infections from health claims data (Germanos et al., 2020).

Table S4: ATC Codes used to specify the date of urinary tract infection

**Description**	**ATC Codes**
Doxycyclin	J01AA02
Tetracyclin	J01AA07
Minocyclin	J01AA08
Ampicillin	J01CA01
Amoxicillin	J01CA04
Pivmecillinam	J01CA08
Mezlocillin	J01CA10
Piperacillin	J01CA12
Temocillin	J01CA17
Benzylpenicillin	J01CE01
Benzylpenicillin-Benzathin	J01CE08
Sulbactam	J01CG01
Amoxicillin and Beta- Lactamase-Inhibitors (Ampicillin + Sulbactam)	J01CR01
Amoxicillin and Beta- Lactamase-Inhibitors (Amoxicillin und Clavulansäure)	J01CR02
Sultamicillin	J01CR04
Piperacillin + Beta-Lactamase-Inhibitors (Piperacillin + Tazobactam)	J01CR05
Cefalexin	J01DB01
Cefazolin	J01DB04
Cefadroxil	J01DB05
Cefuroxim (Cefuroximaxetil)	J01DC02
Cefaclor	J01DC04
Cefotaxim	J01DD01
Ceftazidim	J01DD02
Ceftriaxon	J01DD04
Cefixim	J01DD08
Cefpodoxim	J01DD13
Ceftibuten	J01DD14
Ceftazidim + Beta-Lactamase-Inhibitors	J01DD52
Cefepim	J01DE01
Cefepim + Beta-Lactamase-Inhibitors	J01DE51
Meropenem	J01DH02
Ertapenem	J01DH03
Imipenem + Cilastatin	J01DH51
Ceftobiprol medocaril	J01DI01

Ceftarolin fosamil	J01DI02
Cefiderocol	J01DI04
Ceftolozan + Beta-Lactamase-Inhibitors	J01DI54
Trimethoprim	J01EA01
Sulfamethoxazol und Trimethoprim (Cotrimoxazol)	J01EE01
Erythromycin	J01FA01
Roxithromycin	J01FA06
Azithromycin	J01FA10
Tobramycin	J01GB01
Gentamicin	J01GB03
Neomycin	J01GB05
Ofloxacin	J01MA01
Ciprofloxacin	J01MA02
Enoxacin	J01MA04
Norfloxacin	J01MA06
Levofloxacin	J01MA12
Teicoplanin	J01XA02
Telavancin	J01XA03
Colistin	J01XB01
Nitrofurantoin	J01XE01
Nitrofurantoin, Combinations	J01XE51
Fosfomycin	J01XX01
Nitroxolin	J01XX07

3 TREATMENT DEFINITIONS

In Germany, both herpes zoster and influenza vaccinations are associated with different GOP (in german: “Gebührenordnungsposition”) codes, which are used by physicians to get reimbursed for performed vaccinations. These codes differ over time and by federal states. All codes are associated with the exact date on which the vaccination was performed. Below we list all GOP codes that were used to identify the relevant vaccinations in the BARMER data.

3.1 Herpes Zoster Vaccination

Table S5: GOP Codes used to identify herpes zoster vaccinations by federal state and time

Region	GOP	Valid from	Valid to
Brandenburg	89128B	2019-05-01	2022-01-01
Bremen	89128B	2019-05-01	2022-01-01
Hamburg	89128B	2019-05-01	2022-01-01
Mecklenburg-Vorpommern	89128B	2019-05-01	2022-01-01
Nordrhein	89128B	2019-05-01	2022-01-01
Rheinland-Pfalz	89128B	2019-05-01	2022-01-01
Saarland	89128B	2019-05-01	2022-01-01
Thueringen	89128B	2019-05-01	2022-01-01
Westfalen-Lippe	89128B	2019-05-01	2022-01-01
Sachsen-Anhalt	89128B	2019-05-02	2022-01-01
Hessen	89128B	2019-10-01	2022-01-01
Berlin	89128B	2021-04-01	2022-01-01
Rheinland-Pfalz	89128F	2019-05-01	2022-01-01
Rheinland-Pfalz	89128G	2019-05-01	2022-01-01
Rheinland-Pfalz	89128J	2019-05-01	2022-01-01
Rheinland-Pfalz	89128K	2019-05-01	2022-01-01
Berlin	89129	2019-04-01	2021-06-30
Schleswig-Holstein	89129A	2019-01-01	2022-01-01
Niedersachsen	89129A	2019-04-01	2022-01-01
Sachsen	89129A	2019-04-01	2022-01-01
Baden-Wuerttemberg	89129A	2019-05-01	2022-01-01
Bayern	89129A	2019-05-01	2022-01-01
Brandenburg	89129A	2019-05-01	2022-01-01
Bremen	89129A	2019-05-01	2022-01-01
Hamburg	89129A	2019-05-01	2022-01-01
Mecklenburg-Vorpommern	89129A	2019-05-01	2022-01-01
Nordrhein	89129A	2019-05-01	2022-01-01
Rheinland-Pfalz	89129A	2019-05-01	2022-01-01
Saarland	89129A	2019-05-01	2022-01-01
Thueringen	89129A	2019-05-01	2022-01-01
Westfalen-Lippe	89129A	2019-05-01	2022-01-01
Sachsen-Anhalt	89129A	2019-05-02	2022-01-01

Hessen	89129A	2019-10-01	2022-01-01
Berlin	89129A	2021-04-01	2022-01-01
Schleswig-Holstein	89129B	2019-01-01	2022-01-01
Niedersachsen	89129B	2019-04-01	2022-01-01
Sachsen	89129B	2019-04-01	2022-01-01
Baden-Wuerttemberg	89129B	2019-05-01	2022-01-01
Bayern	89129B	2019-05-01	2022-01-01
Brandenburg	89129B	2019-05-01	2022-01-01
Bremen	89129B	2019-05-01	2022-01-01
Hamburg	89129B	2019-05-01	2022-01-01
Mecklenburg-Vorpommern	89129B	2019-05-01	2022-01-01
Nordrhein	89129B	2019-05-01	2022-01-01
Rheinland-Pfalz	89129B	2019-05-01	2022-01-01
Saarland	89129B	2019-05-01	2022-01-01
Thueringen	89129B	2019-05-01	2022-01-01
Westfalen-Lippe	89129B	2019-05-01	2022-01-01
Sachsen-Anhalt	89129B	2019-05-02	2022-01-01
Hessen	89129B	2019-10-01	2022-01-01
Berlin	89129B	2021-04-01	2022-01-01
Rheinland-Pfalz	89129F	2019-05-01	2022-01-01
Rheinland-Pfalz	89129G	2019-05-01	2022-01-01
Rheinland-Pfalz	89129J	2019-05-01	2022-01-01
Rheinland-Pfalz	89129K	2019-05-01	2022-01-01
Sachsen	99793	2010-07-01	2022-01-01
Berlin	89128	2019-04-01	2021-06-30
Schleswig-Holstein	89128A	2019-01-01	2022-01-01
Niedersachsen	89128A	2019-04-01	2022-01-01
Sachsen	89128A	2019-04-01	2022-01-01
Baden-Wuerttemberg	89128A	2019-05-01	2022-01-01
Bayern	89128A	2019-05-01	2022-01-01
Brandenburg	89128A	2019-05-01	2022-01-01
Bremen	89128A	2019-05-01	2022-01-01
Hamburg	89128A	2019-05-01	2022-01-01
Mecklenburg-Vorpommern	89128A	2019-05-01	2022-01-01
Nordrhein	89128A	2019-05-01	2022-01-01
Rheinland-Pfalz	89128A	2019-05-01	2022-01-01
Saarland	89128A	2019-05-01	2022-01-01
Thueringen	89128A	2019-05-01	2022-01-01
Westfalen-Lippe	89128A	2019-05-01	2022-01-01
Sachsen-Anhalt	89128A	2019-05-02	2022-01-01
Hessen	89128A	2019-10-01	2022-01-01
Berlin	89128A	2021-04-01	2022-01-01
Schleswig-Holstein	89128B	2019-01-01	2022-01-01
Niedersachsen	89128B	2019-04-01	2022-01-01
Sachsen	89128B	2019-04-01	2022-01-01

Baden-Wuerttemberg	89128B	2019-05-01	2022-01-01
Bayern	89128B	2019-05-01	2022-01-01

3.2 Influenza Vaccination

Table S6: GOP Codes used to identify influenza vaccinations by federal state and time

**Region**	**GOP**	**Valid from**	**Valid to**
Sachsen-Anhalt	89004	2005-04-01	2013-03-31
Sachsen-Anhalt	89004F	2005-04-01	2013-03-31
Sachsen-Anhalt	89004G	2005-04-01	2013-03-31
Sachsen-Anhalt	89004M	2005-04-01	2013-03-31
Bayern	89111	2005-04-01	2022-01-01
Baden-Wuerttemberg	89111	2008-01-01	2022-01-01
Berlin	89111	2008-01-01	2022-01-01
Rheinland-Pfalz	89111	2008-01-01	2022-01-01
Sachsen	89111	2008-01-01	2022-01-01
Hessen	89111	2008-07-01	2022-01-01
Mecklenburg-Vorpommern	89111	2008-07-01	2022-01-01
Niedersachsen	89111	2008-07-01	2022-01-01
Nordrhein	89111	2008-07-01	2022-01-01
Saarland	89111	2008-07-01	2022-01-01
Schleswig-Holstein	89111	2008-07-01	2022-01-01
Thueringen	89111	2008-07-01	2022-01-01
Westfalen-Lippe	89111	2008-10-01	2022-01-01
Brandenburg	89111	2009-01-01	2022-01-01
Bremen	89111	2009-01-01	2022-01-01
Hamburg	89111	2012-01-01	2022-01-01
Sachsen-Anhalt	89111	2013-04-01	2022-01-01
Rheinland-Pfalz	89111F	2009-01-01	2022-01-01
Nordrhein	89111H	2008-07-01	2014-12-31
Rheinland-Pfalz	89111J	2012-04-01	2022-01-01
Sachsen	89111S	2008-01-01	2022-01-01
Hamburg	89111S	2020-10-01	2022-01-01
Sachsen	89111X	2014-10-01	2020-09-30
Bayern	89112	2005-04-01	2022-01-01
Baden-Wuerttemberg	89112	2008-01-01	2022-01-01
Rheinland-Pfalz	89112	2008-01-01	2022-01-01
Saarland	89112	2008-01-01	2022-01-01
Sachsen	89112	2008-01-01	2022-01-01
Hessen	89112	2008-07-01	2022-01-01
Mecklenburg-Vorpommern	89112	2008-07-01	2022-01-01
Nordrhein	89112	2008-07-01	2022-01-01
Schleswig-Holstein	89112	2008-07-01	2022-01-01

Thueringen	89112	2008-07-01	2022-01-01
Westfalen-Lippe	89112	2008-10-01	2022-01-01
Brandenburg	89112	2009-01-01	2022-01-01
Bremen	89112	2009-01-01	2022-01-01
Hamburg	89112	2012-01-01	2022-01-01
Sachsen-Anhalt	89112	2013-04-01	2022-01-01
Berlin	89112	2021-04-01	2022-01-01
Bayern	89112B	2020-11-25	2021-06-30
Rheinland-Pfalz	89112F	2009-01-01	2022-01-01
Nordrhein	89112H	2008-07-01	2014-12-31
Rheinland-Pfalz	89112J	2012-04-01	2022-01-01
Rheinland-Pfalz	89112L	2020-01-01	2022-01-01
Brandenburg	89112N	2012-10-01	2019-06-30
Bremen	89112N	2013-01-01	2017-09-30
Westfalen-Lippe	89112N	2013-01-01	2018-03-31
Niedersachsen	89112N	2013-01-01	2018-12-31
Nordrhein	89112N	2013-01-01	2021-09-30
Rheinland-Pfalz	89112N	2013-03-01	2018-03-31
Thueringen	89112N	2013-03-14	2017-05-19
Hamburg	89112N	2013-03-14	2022-01-01
Sachsen	89112N	2013-04-01	2017-12-31
Mecklenburg-Vorpommern	89112N	2013-04-01	2021-03-31
Baden-Wuerttemberg	89112N	2013-04-01	2021-09-30
Sachsen-Anhalt	89112N	2013-04-01	2021-09-30
Schleswig-Holstein	89112N	2013-07-01	2021-09-30
Saarland	89112N	2014-01-01	2021-09-30
Bayern	89112N	2014-09-09	2017-12-31
Hessen	89112N	2015-04-01	2022-01-01
Baden-Wuerttemberg	89112O	2013-10-01	2016-06-30
Rheinland-Pfalz	89112O	2020-01-01	2022-01-01
Sachsen	89112S	2013-01-01	2022-01-01
Baden-Wuerttemberg	89112S	2013-10-01	2016-06-30
Nordrhein	89112T	2009-01-01	2022-01-01
Rheinland-Pfalz	89112V	2013-03-01	2018-09-30
Nordrhein	89112V	2018-01-11	2018-06-30
Rheinland-Pfalz	89112W	2013-03-01	2018-12-31
Baden-Wuerttemberg	89112Y	2019-07-01	2022-01-01
Bayern	89112Y	2019-12-28	2022-01-01
Sachsen-Anhalt	89112Y	2019-12-28	2022-01-01
Thueringen	89112Y	2019-12-28	2022-01-01
Hamburg	89112Y	2020-01-01	2022-01-01
Hessen	89112Y	2020-01-01	2022-01-01
Mecklenburg-Vorpommern	89112Y	2020-01-01	2022-01-01
Nordrhein	89112Y	2020-01-01	2022-01-01
Rheinland-Pfalz	89112Y	2020-01-01	2022-01-01

Sachsen	89112Y	2020-01-01	2022-01-01
Schleswig-Holstein	89112Y	2020-01-01	2022-01-01
Brandenburg	89112Y	2020-04-01	2022-01-01
Westfalen-Lippe	89112Y	2020-08-15	2022-01-01
Niedersachsen	89112Y	2020-09-01	2022-01-01
Bremen	89112Y	2020-10-01	2022-01-01
Berlin	89112Y	2021-04-01	2022-01-01
Bayern	89112Z	2008-10-01	2022-01-01
Baden-Wuerttemberg	89133	2008-01-01	2022-01-01
Hamburg	89731	2018-01-19	2018-12-31
Hessen	97002	2008-01-01	2022-01-01
Bayern	97031C	2018-01-01	2022-01-01
Hessen	97035	2009-01-01	2022-01-01
Rheinland-Pfalz	98880	2011-09-01	2021-03-31
Schleswig-Holstein	99063M	2012-10-01	2014-03-31
Schleswig-Holstein	99063N	2012-10-01	2014-03-31
Schleswig-Holstein	99063O	2012-10-01	2014-03-31

4 DISEASE DEFINITIONS

A case definition for all three autoimmune diseases of interest multiple sclerosis (MS), chronic inflammatory rheumatic disease (CIRD) and chronic inflammatory bowel disease (ger.: Chronisch entzündliche Darmerkrankungen (IBD)) (short form for the three diseases: MS-Athritis-Colitis (MAC)) was developed to identify insurants with prevalent MAC from the claims data. The case definitions were co-designed with clinicians in an iterative process. First, existing case definitions were identified from international literature. In a second step, amalgamated definitions (up to three per condition) were developed based on the literature findings. In a third step, a series of online workshops with data analysts and clinicians was held, focused on the clinical relevance and transferability to the German health care sector. At the end of this process, the final case definitions for the identification of MAC patients in German claims data were established. The final definitions for each of the three conditions are described in the following.

4.1 MS

The ICD-10 diagnoses used for the case definition are displayed in table S7.

Table S7. ICD-10 diagnoses according to ICD10-GM for an MS disease

ICD-10 Code	Description
G35.0	First manifestation of multiple sclerosis
G35.1	Multiple sclerosis with predominantly relapsing course
G35.2	Multiple sclerosis with primary chronic course
G35.3	Multiple sclerosis with secondary chronic course
G35.9	Multiple sclerosis, unspecified

The final case definition for MS is based on a definition developed by Culpepper et al. (2019). The following three criteria are used and combined for a prevalent case of a MS patient as defined below:

- An inpatient main or secondary discharge diagnosis in one quarter [IP]
- A secured diagnosis by a German primary care physician (German primary care in the sense of general practitioners (GP)s and specialists working in private practice) in one quarter [AC]
- A prescription for a disease modifying therapy (DMT) for MS patients in the same year (see table S7). [DMT]

Prevalent MS patients are defined according to the following structure:

(AC **OR** IP) **AND** (AC **OR** IP **OR** DMT) **AND** (AC **OR** IP **OR** DMT)

A visualization of the case definition is given in figure S1.

As one can see in figure S1, it does not count if one patient has only three prescriptions of DMT for MS without at least one diagnosis for MS. This specification was made since some drugs are not used exclusively for the treatment of MS patients. Further specifications were made for IP and AC. If an inpatient and a diagnosis by a primary care physician existed in the same quarter, it was counted as two diagnoses. Likewise, two diagnoses within one quarter were counted individually if two different physicians documented the diagnosis.

Figure S1. Case definition for MS

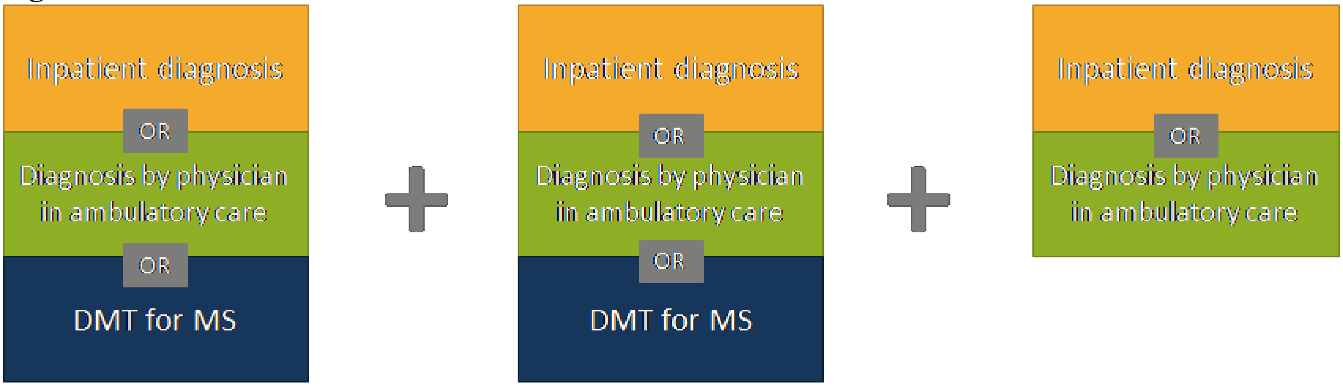

Table S8. Relevant drugs for patients with MS

ATC 2022	OPS[a]	Substance
J06BA01		immunoglobulins, normal human, for extravascular adm.
J06BA02		immunoglobulins, normal human, for intravascular adm.
L01AA01		cyclophosphamide
L01DB07		mitoxantrone
L01FA01	6-001.6[b], 6-001.h[c], 6-001.j[c]	rituximab
L03AB07		interferon beta-1a
L03AB08		interferon beta-1b
L03AB13		peginterferon beta-1a
L03AX13		glatiramer acetate
L04AA23	6-003.f[d]	natalizumab
L04AA27		fingolimod
L04AA36	6-00a.e[e]	ocrelizumab
L04AA40, L01BB04	6-00a.4[e]	cladribine
L04AX01		azathioprine
L04AA31		teriflunomide
L04AA34	6-001.0[d]	alemtuzumab
L04AA42		siponimod
L04AC01	6-009.9[f]	daclizumab
L04AX07, D05BX02		dimethyl fumarate
L04AX09		diroximel fumarate
L04AA52, L01FA02	6-006.4[d]	ofatumumab
L04AA50		ponesimod
L04AA38		ozanimod

[a]Operation and procedure code [ger.: Operationen- und Prozedurenschlüssel (OPS)], chapter 6 Drugs;
[b]Period of validity: 2013–2014
[c]Period of validity: 2015–2021
[d]Period of validity: 2013–2021
[e]Period of validity: 2019–2021
[f]Period of validity: 2018–2021

4.2 CIRD

Due to the high prevalence of chronic inflammatory rheumatic disease, it was decided to specifically address three rheumatic diseases to represent the overall CIRD population: rheumatoid arthritis (RA), axial spondyloarthritis (axSpa) and systemic lupus erythematosus (SLE). For each of the chosen diseases we consented a specific case definition.

4.2.1 RA

For RA, a definition was identified from a German study on the prevalence of RA using statutory health insurance claims data (Hense et al., 2016). We based our case definition for RA on the definitions described in this paper. The definitions were supplemented by discussions with the clinicians of the project. Our final case definition is made up of three options. The chosen ICD-Codes for RA are listed in table S9.

Table S9. ICD-10-Codes for RA

ICD-10 Code	Description
**M05.- Seropositive rheumatoid arthritis**	
M05.0	Felty syndrome
M05.1	Rheumatoid lung disease
M05.2	Rheumatoid vasculitis
M05.3	Rheumatoid arthritis with involvement of other organs and systems
M05.8	Other seropositive rheumatoid arthritis
M05.9	Seropositive rheumatoid arthritis, unspecified
**M06.- Other rheumatoid arthritis**	
M06.0	Seronegative rheumatoid arthritis
M06.1	Adult-onset Still disease
M06.2	Rheumatoid bursitis
M06.3	Rheumatic nodule
M06.4	Inflammatory polyarthropathy
M06.8	Other specified rheumatoid arthritis
M06.9	Rheumatoid arthritis, unspecified

The three options to define a RA in our dataset are the following:

1.) (An inpatient main discharge diagnosis in one quarter **OR** (two inpatient secondary diagnosis **OR** a secured diagnosis by a German primary care physician in two different quarters within one year) **AND** (at least one determination of the C-reactive protein (CRP) **OR** at least one determination of the erythrocyte sedimentation rate (ESR) in ambulatory care (see table S10) within the same year)

OR

2.) (An inpatient main discharge diagnosis in one quarter **OR** (two inpatient secondary diagnosis **OR** an secured diagnosis by a German primary care physician in two different quarters within one year) **AND** at least one prescription of a disease-modifying antirheumatic drug (except glucocorticoids) (see table S11)

OR

3.) (An inpatient main discharge diagnosis in one quarter **OR** (two inpatient secondary diagnosis **OR** an secured diagnosis by a German primary care physician of which at least one is documented by a rheumatologist in two different quarters within one year) **AND** prescription of a glucocorticoid (see table S11) within the same year

Table S10. Relevant EBM (in german: “Einheitlicher Bewertungsmaßstab”) for RA

EBM	Description
32042	Determination of the erythrocyte sedimentation rate
32128	Immunological or equivalent chemical detection of C-reactive protein
32460	Quantitative determination by immunonephelometry, immunoturbidimetry, immunoprecipitation, immunoassay or other equivalent methods of the C-reactive protein (CRP)

Table S11. Relevant Medication for RA

ATC in 2022	OPS[a]	Substance	Type
M01CB03		auranofin	antirheumatic
L04AB05	6-005.7[d]	certolizumab pegol	antirheumatic
H02AB03		fluocortolone	glucocorticoids
M01CB01		sodium aurothiomalate	antirheumatic
L04AA24	6-003.m[b], 6-003.s[c], 6-003.t[c]	abatacept	antirheumatic
L04AB04	6-001.d[d]	adalimumab	antirheumatic
L04AC03		anakinra	antirheumatic
L04AX01		azathioprine	antirheumatic
L04AA37		baricitinib	antirheumatic
H02AB01, H02AB51, H02BX09		betamethasone	glucocorticoids
P01BA01		chloroquine	antirheumatic
L04AD01		ciclosporin	antirheumatic
H02AB02, H02BX02		dexamethasone	glucocorticoids
L04AB01	6-002.b[d]	etanercept	antirheumatic
L04AA45		filgotinib	antirheumatic
L04AB06	6-005.2[d]	golimumab	antirheumatic
P01BA02		hydroxychloroquine	antirheumatic
L04AB02	6-001.e[d]	infliximab	antirheumatic
L04AA13		leflunomide	antirheumatic
L01BA01, L04AX03, M01CX01		methotrexate	antirheumatic
H02AB04, H02AB54, H02BX01		methylprednisolone	glucocorticoids
M01CC01		penicillamine	antirheumatic
H02AB06, H02AB56, H02BX06		prednisolone	glucocorticoids
H02AB07		prednisone	glucocorticoids
L01FA01	6-001.6[b], 6-001.h[c] 6-001.j[c]	rituximab	antirheumatic
L04AC14	6-00a.g[h]	sarilumab	antirheumatic
A07EC01, M01CX02		sulfasalazine	antirheumatic
L04AD02	8-83b.09[d]	tacrolimus	antirheumatic
L04AC07	6-005.3[e], 6-005.m[f], 6-005.n[g]	tocilizumab	antirheumatic
L04AA29		tofacitinib	antirheumatic
L04AA44		upadacitinib	antirheumatic

[a]Operation and procedure code [ger.: Operationen- und Prozedurenschlüssel (OPS)], chapter 6 Drugs; [b]Period of validity: 2013–2014
[c]Period of validity: 2015–2021
[d]Period of validity: 2013–2021
[e]Period of validity 2013–2015
[f]Period of validity: 2016–2021
[g]Period of validity: 2017–2021
[h]Period of validity: 2019–2021

4.2.2 axSpa

Existing definitions for axSpa could be identified from various publications (Blaschke et al., 2021; Krüger et al., 2018; Sloan et al., 2019). To these, further criteria were again added in discussion with the clinicians of the project to arrive at a definition based on three options. We use the ICD-10 codes listed in table S12 with the following criteria:

1.) One inpatient main discharge diagnosis

OR

2.) Two secured diagnosis by a German primary care physician or two inpatient secondary diagnosis in two different quarters within two years

OR

3.) At least one secured diagnosis by a physician in ambulatory care or secondary inpatient diagnosis **AND** one prescription of Non-steroidal anti-inflammatory drugs and anti-rheumatic drugs (ger.: Nichtsteroidale Antiphlogistika und Antirheumatika (NSAR), see table S13) within two years.

Table S12. Relevant ICD-10 Codes for axSpa

ICD-10 Code	Description
**M45.- Ankylosing spondylitis**	
M45.0	Ankylosing spondylitis
**M46.- Other inflammatory spondylopathies**	
M46.8	Other specified inflammatory spondylopathies

Table S13. List of included NSAR

ATC Code	Substance Group
M01AA	Butylpyrazolidines
M01AB	Acetic acid derivatives and related substances
M01AC	Oxicams
M01AE	Propionic acid derivatives
M01AH	Coxibs

4.2.3 SLE

We found a publication of Hanly et al. (2014) comparing different case definitions for SLE. After discussions with the project clinicians, the following definition was chosen as the most fitting for the German health care sector. The options for a SLE case definition are:

1.) One inpatient main discharge diagnosis

OR

2.) Two secured diagnosis by German primary care physicians or inpatient secondary diagnosis in two different quarters within one year

Table S14. Relevant ICD-10 Codes for SLE

ICD-10 Code	Description
**M32.- Systemic lupus erythematosus**	
M32.1	Systemic lupus erythematosus with organ or system involvement
M32.8	Other forms of systemic lupus erythematosus
M32.9	Systemic lupus erythematosus, unspecified

4.3 IBD

Similarly, to RA, the two most common chronic inflammatory bowel diseases were chosen to represent the wider IBD patient population. These are Crohn's disease (CD) and Ulcerative colitis (UC). The two are both defined in the same way following the recommendations given in Rezaie et al. (2012) and Morikubo et al. (2021) as one patient counts as a IBD-patient with the following options:

1.) At least two inpatient discharge diagnosis (main or secondary) within two years **AND** at least one specific prescription of a IBD medication

OR

2.) At least two secured diagnosis by a German primary care physician within two years **AND** at least one specific prescription of a IBD medication

If a patient is detected to have both CD and UC, the patient is excluded of our investigation because the patient cannot be reliably assigned to one specific disease and it is implausible to have both.

Table S15. Relevant ICD-10 Codes for Crohn disease

ICD-10 Code	Description
**K50.- Crohn disease**	
K50.0	Crohn disease of small intestine
K50.1	Crohn disease of large intestine
K50.8	Other Crohn disease
K50.9	Crohn disease, unspecified

Table S16. Relevant ICD-10 Codes for Ulcerative colitis

ICD-10 Code	Description
**K51.- Ulcerative colitis**	
K51.0	Ulcerative (chronic) pancolitis
K51.2	Ulcerative (chronic) proctitis
K51.3	Ulcerative (chronic) rectosigmoiditis
K51.4	Inflammatory polyps
K51.5	Left sided colitis
K51.8	Other ulcerative colitis
K51.9	Ulcerative colitis, unspecified

In order to account for possible changes in the ICD-10 Diagnoses and OPS, code inventories for all observation years covered by the study (2013–2021) were reviewed. No changes in the ICD-10 Codes were found. The OPS codes regarding the relevant years are displayed in table S17. In order to include all relevant ATC codes per year under observation, the "Scientific Institute of AOK" [ger.: Wissenschaftliches Institut der AOK (WIdO)] Pharmaceutical Master File [ger.: Arzneimittel-Stammdatei] of 2022 was used. Based on the ATC codes valid in 2022, a list of all existing Pharmaceutical Central Numbers [ger.: Pharmazentralnummern (PZN)] was created, which was used to decode the drugs for each year.

Table S17. Relevant drugs for patients with IBD

ATC 2022	OPS[a]	Substance
L04AB04	6-001.d[b]	adalimumab
L04AX01		azathioprine
H02AB01, H02AB51, H02BX09		betamethasone
A07EA06		budesonide
L04AD01		ciclosporin
H02AB02, H02BX02		dexamethasone
L04AA45		filgotinib
L04AB06	6-005.2[b]	golimumab
A07EA02, H02AB09		hydrocortisone
L04AB02	6-001.e[b]	infliximab
L01BB02		mercaptopurine
A07EC02		mesalazine
L01BA01, L04AX03, M01CX01		methotrexate
L04AA06		mycophenolic acid
L04AA23	6-003.f[b]	natalizumab
A07EC03		olsalazine
H02AB06, H02AB56, H02BX06		prednisolone
H02AB07		prednisone
A07EC01, M01CX02		sulfasalazine
L04AD02	8-83b.09[b]	tacrolimus
L04AA29		tofacitinib
A01AC01, H02AB08, H02AB58		triamcinolone
L04AA44		upadacitinib
L04AC05	6-005.p[b], 6-001.q[b]	ustekinumab
L04AA33	6-008.5[b]	vedolizumab

[a]Operation and procedure code [ger.: Operationen- und Prozedurenschlüssel (OPS)], chapter 6 Drugs;
[b]Period of validity: 2013–2021

5 OTHER DEFINITIONS

Below we give further definitions for some variables that will be used to adjust for confounding in the proposed analyses.

5.1 Pneumococcal Vaccination

The timing of pneumococcal vaccination will be determined using GOP codes, as previously described for the influenza and herpes zoster vaccinations. The relevant GOP codes are listed in table S18.

Table S18: GOP Codes used to identify herpes zoster vaccinations by federal state and time

Region	GOP	Valid from	Valid to
Sachsen-Anhalt	89014F	2005-04-01	2013-03-31
Sachsen-Anhalt	89014G	2005-04-01	2013-03-31
Sachsen-Anhalt	89014M	2005-04-01	2013-03-31
Berlin	89118	2008-01-01	2021-06-30
Baden-Wuerttemberg	89118A	2008-01-01	2022-01-01
Niedersachsen	89118A	2008-01-01	2022-01-01
Rheinland-Pfalz	89118A	2008-01-01	2022-01-01
Sachsen	89118A	2008-01-01	2022-01-01
Hessen	89118A	2008-07-01	2022-01-01
Mecklenburg-Vorpommern	89118A	2008-07-01	2022-01-01
Nordrhein	89118A	2008-07-01	2022-01-01
Saarland	89118A	2008-07-01	2022-01-01
Schleswig-Holstein	89118A	2008-07-01	2022-01-01
Thueringen	89118A	2008-07-01	2022-01-01
Bayern	89118A	2008-10-01	2022-01-01
Westfalen-Lippe	89118A	2008-10-01	2022-01-01
Brandenburg	89118A	2009-01-01	2022-01-01
Bremen	89118A	2009-01-01	2022-01-01
Hamburg	89118A	2012-01-01	2022-01-01
Sachsen-Anhalt	89118A	2013-04-01	2022-01-01
Berlin	89118A	2021-04-01	2022-01-01
Baden-Wuerttemberg	89118B	2008-01-01	2022-01-01
Niedersachsen	89118B	2008-01-01	2022-01-01
Rheinland-Pfalz	89118B	2008-01-01	2022-01-01
Sachsen	89118B	2008-01-01	2022-01-01
Hessen	89118B	2008-07-01	2022-01-01
Mecklenburg-Vorpommern	89118B	2008-07-01	2022-01-01
Nordrhein	89118B	2008-07-01	2022-01-01
Saarland	89118B	2008-07-01	2022-01-01
Schleswig-Holstein	89118B	2008-07-01	2022-01-01
Thueringen	89118B	2008-07-01	2022-01-01
Bayern	89118B	2008-10-01	2022-01-01

Westfalen-Lippe	89118B	2008-10-01	2022-01-01
Brandenburg	89118B	2009-01-01	2022-01-01
Bremen	89118B	2009-01-01	2022-01-01
Hamburg	89118B	2012-01-01	2022-01-01
Sachsen-Anhalt	89118B	2013-04-01	2022-01-01
Berlin	89118B	2021-04-01	2022-01-01
Rheinland-Pfalz	89118F	2009-01-01	2022-01-01
Rheinland-Pfalz	89118G	2009-01-01	2022-01-01
Nordrhein	89118H	2008-07-01	2014-12-31
Rheinland-Pfalz	89118J	2012-04-01	2022-01-01
Rheinland-Pfalz	89118K	2012-04-01	2022-01-01
Bayern	89119	2007-04-01	2022-01-01
Berlin	89119	2008-01-01	2022-01-01
Rheinland-Pfalz	89119	2008-01-01	2022-01-01
Hessen	89119	2008-07-01	2022-01-01
Mecklenburg-Vorpommern	89119	2008-07-01	2022-01-01
Niedersachsen	89119	2008-07-01	2022-01-01
Nordrhein	89119	2008-07-01	2022-01-01
Schleswig-Holstein	89119	2008-07-01	2022-01-01
Thueringen	89119	2008-07-01	2022-01-01
Baden-Wuerttemberg	89119	2008-10-01	2022-01-01
Sachsen	89119	2008-10-01	2022-01-01
Westfalen-Lippe	89119	2008-10-01	2022-01-01
Brandenburg	89119	2009-01-01	2022-01-01
Bremen	89119	2009-01-01	2022-01-01
Saarland	89119	2009-01-01	2022-01-01
Hamburg	89119	2012-01-01	2022-01-01
Sachsen-Anhalt	89119	2013-04-01	2022-01-01
Rheinland-Pfalz	89119F	2009-01-01	2022-01-01
Nordrhein	89119H	2008-07-01	2014-12-31
Rheinland-Pfalz	89119J	2012-04-01	2022-01-01
Bremen	89119R	2017-01-01	2022-01-01
Sachsen-Anhalt	89119R	2017-01-01	2022-01-01
Thueringen	89119R	2017-01-01	2022-01-01
Brandenburg	89119R	2017-04-01	2022-01-01
Niedersachsen	89119R	2017-04-01	2022-01-01
Bayern	89119R	2017-05-19	2022-01-01
Hamburg	89119R	2017-05-19	2022-01-01
Rheinland-Pfalz	89119R	2017-05-19	2022-01-01
Saarland	89119R	2017-05-19	2022-01-01
Baden-Wuerttemberg	89119R	2017-05-20	2022-01-01
Hessen	89119R	2017-05-20	2022-01-01
Sachsen	89119R	2017-05-20	2022-01-01
Mecklenburg-Vorpommern	89119R	2017-07-01	2022-01-01
Schleswig-Holstein	89119R	2017-07-01	2022-01-01

Westfalen-Lippe	89119R	2018-01-01	2022-01-01
Nordrhein	89119R	2020-01-01	2022-01-01
Berlin	89119R	2021-04-01	2022-01-01
Rheinland-Pfalz	89119S	2017-05-19	2022-01-01
Rheinland-Pfalz	89119T	2017-05-19	2022-01-01
Bayern	89120	2007-04-01	2022-01-01
Hamburg	89120	2007-04-01	2022-01-01
Baden-Wuerttemberg	89120	2008-01-01	2022-01-01
Bremen	89120	2008-01-01	2022-01-01
Rheinland-Pfalz	89120	2008-01-01	2022-01-01
Mecklenburg-Vorpommern	89120	2008-07-01	2022-01-01
Niedersachsen	89120	2008-07-01	2022-01-01
Nordrhein	89120	2008-07-01	2022-01-01
Schleswig-Holstein	89120	2008-07-01	2022-01-01
Thueringen	89120	2008-07-01	2022-01-01
Sachsen	89120	2008-10-01	2022-01-01
Westfalen-Lippe	89120	2008-10-01	2022-01-01
Brandenburg	89120	2009-01-01	2022-01-01
Sachsen-Anhalt	89120	2013-04-01	2022-01-01
Hessen	89120	2016-01-01	2022-01-01
Berlin	89120	2021-04-01	2022-01-01
Hessen	89120A	2008-07-01	2017-12-31
Rheinland-Pfalz	89120F	2009-01-01	2022-01-01
Nordrhein	89120H	2008-07-01	2014-12-31
Rheinland-Pfalz	89120J	2012-04-01	2022-01-01
Rheinland-Pfalz	89120L	2020-01-01	2022-01-01
Rheinland-Pfalz	89120O	2020-01-01	2022-01-01
Baden-Wuerttemberg	89120R	2008-01-01	2022-01-01
Rheinland-Pfalz	89120R	2008-01-01	2022-01-01
Sachsen	89120R	2008-01-01	2022-01-01
Hessen	89120R	2008-07-01	2022-01-01
Mecklenburg-Vorpommern	89120R	2008-07-01	2022-01-01
Niedersachsen	89120R	2008-07-01	2022-01-01
Nordrhein	89120R	2008-07-01	2022-01-01
Saarland	89120R	2008-07-01	2022-01-01
Schleswig-Holstein	89120R	2008-07-01	2022-01-01
Thueringen	89120R	2008-07-01	2022-01-01
Bayern	89120R	2008-10-01	2022-01-01
Westfalen-Lippe	89120R	2008-10-01	2022-01-01
Brandenburg	89120R	2009-01-01	2022-01-01
Bremen	89120R	2009-01-01	2022-01-01
Hamburg	89120R	2012-01-01	2022-01-01
Sachsen-Anhalt	89120R	2013-04-01	2022-01-01
Berlin	89120R	2021-04-01	2022-01-01
Rheinland-Pfalz	89120S	2009-01-01	2022-01-01

Sachsen	89120S	2012-10-01	2017-06-30
Rheinland-Pfalz	89120T	2012-04-01	2022-01-01
Rheinland-Pfalz	89120U	2020-01-01	2022-01-01
Baden-Wuerttemberg	89120V	2019-07-01	2022-01-01
Bayern	89120V	2019-12-28	2022-01-01
Sachsen-Anhalt	89120V	2019-12-28	2022-01-01
Thueringen	89120V	2019-12-28	2022-01-01
Hamburg	89120V	2020-01-01	2022-01-01
Hessen	89120V	2020-01-01	2022-01-01
Mecklenburg-Vorpommern	89120V	2020-01-01	2022-01-01
Nordrhein	89120V	2020-01-01	2022-01-01
Rheinland-Pfalz	89120V	2020-01-01	2022-01-01
Sachsen	89120V	2020-01-01	2022-01-01
Schleswig-Holstein	89120V	2020-01-01	2022-01-01
Brandenburg	89120V	2020-04-01	2022-01-01
Westfalen-Lippe	89120V	2020-08-15	2022-01-01
Bremen	89120V	2020-10-01	2022-01-01
Niedersachsen	89120V	2021-01-01	2022-01-01
Berlin	89120V	2021-04-01	2022-01-01
Baden-Wuerttemberg	89120X	2019-07-01	2022-01-01
Bayern	89120X	2019-12-28	2022-01-01
Sachsen-Anhalt	89120X	2019-12-28	2022-01-01
Thueringen	89120X	2019-12-28	2022-01-01
Hamburg	89120X	2020-01-01	2022-01-01
Hessen	89120X	2020-01-01	2022-01-01
Mecklenburg-Vorpommern	89120X	2020-01-01	2022-01-01
Nordrhein	89120X	2020-01-01	2022-01-01
Rheinland-Pfalz	89120X	2020-01-01	2022-01-01
Sachsen	89120X	2020-01-01	2022-01-01
Schleswig-Holstein	89120X	2020-01-01	2022-01-01
Brandenburg	89120X	2020-04-01	2022-01-01
Westfalen-Lippe	89120X	2020-08-15	2022-01-01
Bremen	89120X	2020-10-01	2022-01-01
Niedersachsen	89120X	2021-01-01	2022-01-01
Berlin	89120X	2021-04-01	2022-01-01
Rheinland-Pfalz	89120Z	2020-01-01	2022-01-01
Hessen	97013	2008-01-01	2022-01-01
Bayern	97031F	2018-01-01	2022-01-01

5.2 Official Level of Care

Because the official level of care is required for billing purposes, it is included in the BARMER data with exact dates for every change that occurs for every individual. However, the scale itself was changed on the first january of 2017, which necessitates some further discussion. Until the first january of 2017, the level of care was measured on the “Pflegegrad” scale. This scale included three point, with higher levels indicating higher levels of care needed. Since the first january 2017, however, a new scale with five points is used instead. Again, higher levels on the new scale represent higher levels of care. To determine the value on the new “Pflegegrad” scale from the old “Pflegestufe” scale, the value of the old scale is required in addition to knowledge about whether a person has limited everyday competence. A mapping of old to new values is given in table S19.

Table S19. Mapping of Levels in on the Pflegestufe scale to levels in the Pflegegrad scale

(old) Pflegestufe	Limited everyday compete-nce	(new) Pflegegrad
None	no	None
None	yes	1
I	no	2
I	yes	3
II	no	3
II	yes	4
III	no	4
III	yes	5
III (hardship case, in german: “Härtefall”)	not relevant	5

In order to use the level of care as an adjustment variable in the analysis, we have to map the old values on the “Pflegestufe” scale to values on the “Pflegegrad” scale for all times before the first january 2017. For this purpose, we will try to determine whether an individual has limited everyday competence based on ICD-10 codes. The codes shown in table S20 will be used for this purpose.

Table S20. ICD-10 Codes used to determine limited everyday competence

ICD-10 Codes	Description
F00–F09	Organic, including symptomatic mental disorders
F10–F19	Mental and behavioral disorders caused by psychotropic substances
F20–F29	Schizophrenia, schizotopic and delusional disorders
F30–F39	Affective disorders
F40–F49	Neurotic, stress and somatoform disorders
F50–F59	Behavioral problems with physical disorders and factors
F60–F69	Personality and behavioral disorders
F70–F79	Intelligence disorder
F80–F89	Developmental disorders
F90–F98	Behavioral and emotional disorders with onset in childhood and adolescence
F99	Unspecified disorders
Q99	Down-Syndrome

Because ICD-10 codes are not directly associated with specific dates, we will determine limited everyday competence only on a quarterly basis. This information is then used to re-code the old “Pflegestufe” into the new “Pflegegrad” whenever neccessary. The date associated with the “Pflegestufe” will, however, be retained and used in the analysis as is. Because none of the codes used in this definition are acute and usually are not suspect to sudden changes, this method should not lead to any substantial misclassification.

5.3 Chickenpox

The presence of chickenpox, as required in the herpes zoster vaccine effectiveness analysis as a confounder, is determined by the presence or absence of the ICD-10 code **B01**. The first date of the quarter in which this ICD-10 code occurs will be considered the date of onset of this infection.

5.4 Modified Charlson Comorbidity Index

Because one of the main diseases of interest in this study are rheumatic diseases, a modified version of the Charlson comorbidity index (CCI) is calculated in which the rheumatic diseases are not included. Table S21 shows the ICD-10 code based groups that were included in this modified version and how many points are associated with this group. Table S22 shows all ICD-10 codes that were considered for each of those groups. The points of every group that a patient belongs to at the respective point in time are added up to obtain the final modified CCI score. Because the ICD-10 codes are not neccessarily associated with exact dates, the presence of the relevant ICD-10 codes will only be assessed on a quarterly basis. The modified CCI will then be calculated using a rolling time window of a year, meaning that for every quarter the 4 quarters before it will be used to calculate the ”current” CCI. This way, only information about the past is used.

Table S21. Categories and associated points used to calculate the modified Charlson Comorbidity Index

ICD-10 Group	Description	Points
A	Myocardial infarction	1
B	Heart failure	1
C	Peripheral vascular diseases	1
D	Cerebral vascular event	1
E	Dementia	1
F	Chronic pulmonary disease	1
G	Connective tissue disease	1
H	Peptic ulcer	1
Q	Mild liver disease	1
M	Diabetes without chronic complications	1
K	Hemiplegia or paraplegia	2
L	Kidney disease	2
M	Diabetes with complications	2
N	Cancer	2
O	Leukaemia	2
P	Lymphoma	2
Q	Severe liver disease	3
R	Metastasised cancer	6
S	AIDS/HIV positive	6

Table S22: Classification of ICD-10 Codes into categories used in calculation of the modified Charlson Comorbidity Index

ICD-10 Group	ICD-10 code
A	I21
A	I22
A	I25.2
B	I09.9
B	I11.0
B	I13.0
B	I13.2
B	I25.5
B	I42.0
B	I42.5
B	I42.6
B	I42.7
B	I42.8
B	I42.9
B	I43
B	I50
B	P29.0
C	I70
C	I71
C	I73.1
C	I73.8
C	I73.9
C	I77.1
C	I79.0
C	I79.2
C	K55.1
C	K55.8
C	K55.9
C	Z95.8
C	Z95.9
D	G45
D	G46
D	H34.0
D	I60
D	I61
D	I62
D	I63
D	I64
D	I65
D	I66

D	I67
D	I68
D	I69
E	F00
E	F01
E	F02
E	F03
E	F05.1
E	G30
E	G31.1
F	I27.8
F	I27.9
F	J40
F	J41
F	J42
F	J43
F	J44
F	J45
F	J46
F	J47
F	J60
F	J61
F	J62
F	J63
F	J64
F	J65
F	J66
F	J67
F	J68.4
F	J70.1
F	J70.3
G	M31.5
G	M33
G	M34
G	M35.1
G	M35.3
G	M36.0
H	K25
H	K26
H	K27
H	K28
I	B18
I	K70.0
I	K70.1
I	K70.2

I	K70.3
I	K70.9
I	K71.3
I	K71.4
I	K71.5
I	K71.7
I	K73
I	K74
I	K76.0
I	K76.2
I	K76.3
I	K76.4
I	K76.8
I	K76.9
I	Z94.4
J	E10.0
J	E10.1
J	E10.9
J	E11.0
J	E11.1
J	E11.9
J	E12.0
J	E12.1
J	E12.9
J	E13.0
J	E13.1
J	E13.9
J	E14.0
J	E14.1
J	E14.9
K	G04.1
K	G11.4
K	G80.1
K	G80.2
K	G81
K	G82
K	G83.0
K	G83.1
K	G83.2
K	G83.3
K	G83.4
K	G83.9
L	I12.0
L	I13.1
L	N03.2

L	N03.3
L	N03.4
L	N03.5
L	N03.6
L	N03.7
L	N05.2
L	N05.3
L	N05.4
L	N05.5
L	N05.6
L	N05.7
L	N18
L	N19
L	N25
L	Z49.0
L	Z49.1
L	Z49.2
L	Z94.0
L	Z99.2
M	E142
M	E10.2
M	E10.3
M	E10.4
M	E10.5
M	E10.6
M	E10.7
M	E10.8
M	E11.2
M	E11.3
M	E11.4
M	E11.5
M	E11.6
M	E11.7
M	E11.8
M	E12.2
M	E12.3
M	E12.4
M	E12.5
M	E12.6
M	E12.7
M	E12.8
M	E13.2
M	E13.3
M	E13.4
M	E13.5

M	E13.6
M	E13.7
M	E13.8
M	E14.2
M	E14.3
M	E14.4
M	E14.5
M	E14.6
M	E14.7
M	E14.8
N	C00
N	C01
N	C02
N	C03
N	C04
N	C05
N	C06
N	C07
N	C08
N	C09
N	C10
N	C11
N	C12
N	C13
N	C14
N	C15
N	C16
N	C17
N	C18
N	C19
N	C20
N	C21
N	C22
N	C23
N	C24
N	C25
N	C26
N	C30
N	C31
N	C32
N	C33
N	C34
N	C37
N	C38
N	C39

N	C40
N	C41
N	C43
N	C45
N	C46
N	C47
N	C48
N	C49
N	C50
N	C51
N	C52
N	C53
N	C54
N	C55
N	C56
N	C57
N	C58
N	C60
N	C61
N	C62
N	C63
N	C64
N	C65
N	C66
N	C67
N	C68
N	C69
N	C70
N	C71
N	C72
N	C73
N	C74
N	C75
N	C76
N	C97
O	C91
O	C92
O	C93
O	C94
O	C95
P	C81
P	C82
P	C83
P	C84
P	C85

P	C88
P	C90
P	C96
Q	I85.0
Q	I85.9
Q	I86.4
Q	I98.2
Q	K70.4
Q	K71.1
Q	K72.1
Q	K72.9
Q	K76.5
Q	K76.6
Q	K76.7
R	C77
R	C78
R	C79
R	C80
S	B20
S	B21
S	B22
S	B24

5.5 Medical Treatment Classification

A wide range of medications is used to treat patients with either MS, IBD and CIRD. As described in the main text, these medications may have different immunosuppressive effects and should therefore be accounted for in the vaccine effectiveness analyses. Due to the large amount of different relevant medications, it would, however, not be feasible to include the presence or absence of each one as a separate covariate. We therefore classified them into three categories, with regards to the strength of their immunosuppressive effect in regards to the response to vaccination as follows:

1. no immunossuppressive effect
2. mild immunosuppressive effect
3. moderate to severe immunosuppressive effect

The classification was created by a committee of physicians specialized in treatment for MS, IBD and CIRD, based on expert judgement, and, if available, relevant scientific literature. Table S23 shows the resulting classification. ATC codes used to identify the medications are given in multiple tables throughout this supplement. The following relevant medications are missing from this table: prednisolone, hydrocortisone, and triamcinolone. The immunosuppressive effect of these medications is highly dependent on the dosage received and the mode of administration, which might vary a lot for these medications. The classification was therefore made by taking these factors into account. If administered via cutaneous topical application (creams, ointments, and foams), the medication was classified to be level 1 (no immunosuppressive effect). Otherwise, a dosage dependent classification was employed as described in

table S24. Although it might be beneficial to do this for every medication, this was deemed unneccessary due to the relatively small differences in dosage and mode of administration for the other relevant medications.

Table S23: Classification of medical treatments into levels of immunosuppressive effect

Medicine	Level of immunosuppressive effect
Azathioprin	2
Rituximab	2
Natalizumab	2
Immunoglobuline, normal human (extravascular application)	1
Immunoglobuline, normal human (intravascular application)	1
Cyclophosphamide	1
Mitoxantrone	2
Interferon beta-la	1
Interferon beta-lb	1
Peginterferon beta-la	1
Glatirameracetat	1
Fingolimod	2
Ocrelizumab	2
Cladribin	2
Teriflunomid	2
Alemtuzumab	2
Siponimod	2
Daclizumab	2
Dimethylfumarat	2
Diroximelfumarat	2
Ofatumumab	2
Ponesimod	2
Ozanimod	2
Betamethason-Depot	2
Prednisolon-Depot	2
Adalimumab	2
Ciclosporin	2
Dexamethason	2
Filgotinib	2
Golimumab	2
Infliximab	2
Methotrexat	2
Prednison	2
Sulfasalazin	1
Tacrolimus	2
Tofacitinib	2
Upadacitinib	2
Auranofin	1

Certolizumab pegol	2
Fluocortolon	2
Abatacept	2
Anakinra	2
Baricitinib	2
Betamethason	2
Chloroquin	1
Etanercept	2
Hydroxychloroquin	2
Leflunomid	2
Methylprednisolon	2
Sarilumab	2
Tocilizumab	2
Ibupofen (Combinations)	1
Parecoxib	1
Diclofenac (and Omeprazol) (Combinations)	1
Phenylbutazon (Combinations)	1
Tiaprofenic acid	1
Proglumetacin	1
Aceclofenac	1
Dexibuprofen	1
Ketoprofen	1
Naproxen (and Esomeprazol)	1
Piroxicam	1
Acemetacin	1
Dexketoprofen	1
Indometacin	1
Meloxicam	1
Celecoxib	1
Etoricoxib	1
Belimumab	2
Bimekizumab	2
Secukinumab	2
Ixekizumab	2
Mycophenolic acid	2
Budesonid	1
Mercaptopurin	2
Mesalazin	1
Olsalazin	1
Ustekinumab	1
Vedolizumab	1

Table S24. Classification of medical treatments into levels of immunosuppressive effect with dosage dependence

Medicine	Level 2	Level 3
Prednisolone	< 10 mg	≥ 10 mg
Hydrocortisone	< 40 mg	≥ 40 mg
Triamcinolone (Depot)	< 8 mg	≥ 8 mg

In the analyses we will use the exact prescription dates associated with each medical treatment directly. For each day, the highest currently ongoing immunosuppressive effect as defined by the given classification is used for each individual. The immunosuppressive effect has different durations for some medical treatments. The durations we will use from prescription on are given in table S25. These were also discussed by the same committee of expert clinicians.

Table S25: Duration of the immunosuppressive effect of each relevant medical treatment

Medicine	Duration in days
Abatacept	180
Aceclofenac	90
Acemetacin	90
Adalimumab	180
Alemtuzumab	∞
Anakinra	90
Auranofin	270
Azathioprin	90
Baricitinib	90
Belimumab	270
Betamethason	90
Betamethason-Depot	90
Bimekizumab	180
Budesonid	90
Celecoxib	90
Certolizumab pegol	360
Chloroquin	90
Ciclosporin	90
Cladribin	810
Cyclophosphamid	180
Daclizumab	90
Dexamethason	90
Dexibuprofen	90
Dexketoprofen	90
Diclofenac (and Omeprazol) (Combinations)	90
Dimethylfumarat	90
Diroximelfumarat	90
Etanercept	180

Etoricoxib	90
Filgotinib	90
Fingolimod	90
Fluocortolon	90
Glatirameracetat	90
Golimumab	180
Hydrocortison	90
Hydroxychloroquin	90
Ibupofen (Combinations)	90
Immunoglobuline, normal human (extravascular application)	90
Immunoglobuline, normal human (intravascular application)	90
Indometacin	90
Infliximab	180
Interferon beta-la	90
Interferon beta-lb	90
Ixekizumab	180
Ketoprofen	90
Leflunomid	270
Mercaptopurin	90
Meloxicam	90
Mesalazin	90
Methotrexat	180
Methylprednisolon	90
Mitoxantron	90
Mycophenolic acid	90
Naproxen (and Esomeprazol)	90
Natalizumab	90
Ocrelizumab	450
Ofatumumab	90
Olsalazin	90
Ozanimod	90
Parecoxib	90
Peginterferon beta-la	90
Phenylbutazon (Combinations)	90
Piroxicam	90
Ponesimod	90
Prednisolon-Depot	90
Prednisolone	90
Prednison	90
Proglumetacin	90
Rituximab	450
Sarilumab	180
Secukinumab	180
Siponimod	90
Sulfasalazin	90

Tacrolimus	90
Teriflunomid	90
Tiaprofensäure	90
Tocilizumab	180
Tofacitinib	90
Upadacitinib	90
Ustekinumab	180
Vedolizumab	90

REFERENCES

Blaschke, K., Fischer-Betz, R., Marschall, U., Dombrowsky, W., Joeres, L., Heidlbrede, T., et al. (2021). Treatment patterns and resource utilization of pregnant women with inflammatory rheumatic diseases or psoriasis in germany: A claims database analysis. *Rheumatology and Therapy* 8, 1565–1584

Culpepper, W. J., Marrie, R. A., Langer-Gould, A., Wallin, M. T., Campbell, J. D., Nelson, L. M., et al. (2019). Validation of an algorithm for identifying ms cases in administrative health claims datasets. *Neurology* 5, e1016–e1028

Germanos, G., Light, P., Zoorob, R., Salemi, J., Khan, F., Hansen, M., et al. (2020). Validating use of electronic health data to identify patients with urinary tract infections in outpatient settings. *Antibiotics* 9, 1–8

Hanly, J. G., Thompson, K., and Skedgel, C. (2014). Identification of patients with systemic lupus erythematosus in administrative healthcare databases. *Lupus* 23, 1337–1446

Hense, S., Ramos, A. L., Callhoff, J., Albrecht, K., Zink, A., and Hoffmann, F. (2016). Prevalence of rheumatoid arthritis in germany based on health insurance data: Regional differences and first results of the proclair study. *Zeitschrift für Rheumatologie* 75, 819–827

Krüger, K., von Hinüber, U., Meier, F., Tian, H., Böhm, K., Jugl, S. M., et al. (2018). Ankylosing spondylitis causes high burden to patients and the healthcare system: Results from a german claims database analysis. *Rheumatology International* 38, 2121–2131

Morikubo, H., Kobayashi, T., Fukuda, T., Nagahama, T., Hisamatsu, T., and Hibi, T. (2021). Development of algorithms for identifying patients with crohn's disease in japanese health insurance claims database. *PLoS One* 16

Rezaie, A., Quan, H., Fedorak, R. N., Panaccione, R., and Hilsden, R. J. (2012). Development and validation of an administrative case definition for inflammatory bowel diseases. *Canadian Journal of Gastroenterology* 26, 711–717

Sloan, V. S., Sheahan, A., Stark, J. L., and Suruki, R. Y. (2019). Opioid use in patients with ankylosing spondylitis is common in the united states: Outcomes of a retrospective cohort study. *The Journal of Rheumatology* 46, 1450–1457
